# Boosting the activity of Prussian-blue analogue as efficient electrocatalyst for water and urea oxidation

**DOI:** 10.1038/s41598-019-52412-1

**Published:** 2019-11-04

**Authors:** Yongqiang Feng, Xiao Wang, Peipei Dong, Jie Li, Li Feng, Jianfeng Huang, Liyun Cao, Liangliang Feng, Koji Kajiyoshi, Chunru Wang

**Affiliations:** 1 School of Materials Science and Engineering, Shaanxi Key Laboratory of Green Preparation and Functionalization for Inorganic Materials, Key Laboratory of Auxiliary Chemistry and Technology for Chemical Industry, Ministry of Education, Shaanxi University of Science and Technology, Xi’an, 710021 People’s Republic of China; 2 Beijing National Laboratory for Molecular Sciences, Laboratory of Molecular Nanostructure and Nanotechnology, Institute of Chemistry, Chinese Academy of Sciences, Beijing, 100190 China; 3 Research Laboratory of Hydrothermal Chemistry, Faculty of Science and Technology, Kochi University, Kochi, 780-8520 Japan

**Keywords:** Materials for energy and catalysis, Environmental chemistry

## Abstract

The design and fabrication of intricate hollow architectures as cost-effective and dual-function electrocatalyst for water and urea electrolysis is of vital importance to the energy and environment issues. Herein, a facile solvothermal strategy for construction of Prussian-blue analogue (PBA) hollow cages with an open framework was developed. The as-obtained CoFe and NiFe hollow cages (CFHC and NFHC) can be directly utilized as electrocatalysts towards oxygen evolution reaction (OER) and urea oxidation reaction (UOR) with superior catalytic performance (lower electrolysis potential, faster reaction kinetics and long-term durability) compared to their parent solid precursors (CFC and NFC) and even the commercial noble metal-based catalyst. Impressively, to drive a current density of 10 mA cm^−2^ in alkaline solution, the CFHC catalyst required an overpotential of merely 330 mV, 21.99% lower than that of the solid CFC precursor (423 mV) at the same condition. Meanwhile, the NFHC catalyst could deliver a current density as high as 100 mA cm^−2^ for the urea oxidation electrolysis at a potential of only 1.40 V, 24.32% lower than that of the solid NFC precursor (1.85 V). This work provides a new platform to construct intricate hollow structures as promising nano-materials for the application in energy conversion and storage.

## Introduction

Hydrogen energy has been considered as one of the most promising alternatives to traditional fossil fuels such as coal and oil which have inevitably involved in the tough environmental and unsustainable energetic issues^1,2^. Meanwhile, the past decades has witnessed the dramatic development of hydrogen production technologies. For example, electrochemical water splitting (H_2_O → H_2_ + 1/2 O_2_) has attracted much attention, which could produce hydrogen in the cathode and oxygen in the anode, respectively^3–8^. Oxygen evolution reaction (OER) as the half-cell reaction of overall water splitting, however, hinders the water oxidation process due to its sluggish kinetics^9–11^. On the other hand, urea oxidation reaction (UOR) provides much easier accessibility for hydrogen (CO(NH_2_)_2_ + H_2_O → 3H_2_ + CO_2_ + N_2_) owing to the lower thermodynamic potential (0.37 V) for UOR compared with OER (1.23 V)^12,13^. Unfortunately, both OER and UOR require intensively higher energy to drive the reactions in view of the large overpotentials (η) caused during the electrolysis^14^. Although noble-metal based catalysts, i.e. IrO_2_ and RuO_2_, exhibit efficient electrocatalytic performance for OER and UOR, their scarcity and high cost prohibit the large-scale application^15^. Therefore, it is appealing but highly challenging to design and construct earth-abundant and low-cost electrocatalysts for OER and UOR. Up to now, various noble-metal-free catalysts have been developed, such as metal oxides^16,17^, metal hydroxides^18–20^, metal chalcogenides^21,22^, metal phosphides^23^, metal nitrides^24^, and metal carbides^25^, etc. However, the performance of these reported materials as a bifunctional electrocatalyst both for OER and UOR are far from the industrial utilization. Hence, it is of urgent significance to pursue novel efficient dual-function catalyst in combination of OER and UOR application.

Metal-organic frameworks (MOFs) with multivalent transition metals have drawn much attention in many fields, such as solar cells^26^, gas separation^27^, batteries^28^, supercapacitors^29^, biomaterials^30^, hydrogen production^31^, etc. on the merit of their structural tunability and functional versatility^32–37^. In particular, Prussian-blue analogues (PBAs), as one of the prototypical MOF members, have been designated as the pioneer to modulate the structure-dependent catalytic properties^38–44^. Lou *et al*. established a self-templated epitaxial growth strategy for the controllable synthesis of Co-Fe PBA cage, frame and box with diverse geometries, and the frame-like corresponding Co-Fe mixed oxides with retainable architectures exhibited super OER performance^45^. It is illuminating that nanostructured hollow PBAs with more exposed metal sites are prone to be more active during the electrochemical process^46–49^. For example, a Ni-Fe PBA nano-frame structure constructed by preferential etching of PBA cube displayed enhanced specific capacitance and cycling stability when used as the cathode in sodium ion battery (SIB)^50^. Notably, as to the electrocatalytic behaviors for OER and UOR, PBA-based nanostructures are still at a nascent stage. Therefore, it is vital to develop new method for the fabrication of intricate PBA-based architectures used as highly efficient bifunctional electrocatalysts for OER and UOR.

Herein, we built a self-templated three-dimensional (3D) hollow nanocages with an open framework on the corners via a facile solvothermal treatment of the initial Co-Fe PBA cubes (CFC). The *in-situ* released proton from the ethanol solvent induced the structure evolution accompanied with a metal-metal electron transfer (Co^П^-CN-Fe^Ш^ → Co^Ш^-CN-Fe^П^). Furthermore, this strategy was also applicable to Ni-Fe PBA cube (NFC) to construct Ni-Fe hollow cage (NFHC). The obtained Co-Fe hollow cage (CFHC) with larger specific surface area and more active catalytic sites exhibited lower overpotentials and faster charge transfer kinetics both for OER and UOR process. Our work show the feasibility and diversity of template-engaged synthesis of hollow architectures as efficient electrocatalysts, which would shed new light on the design and fabrication of novel promising catalysts with intricate hollow structures to promote the hydrogen production. Moreover, the present method can also pave the way for the construction of novel nano-materials in the realms of energy conversion and storage, such as fuel cells, lithium-ion batteries (LIBs), supercapacitors, etc.

## Results

### Synthesis and characterization of CFHC and NFHC

**Figure 1:**
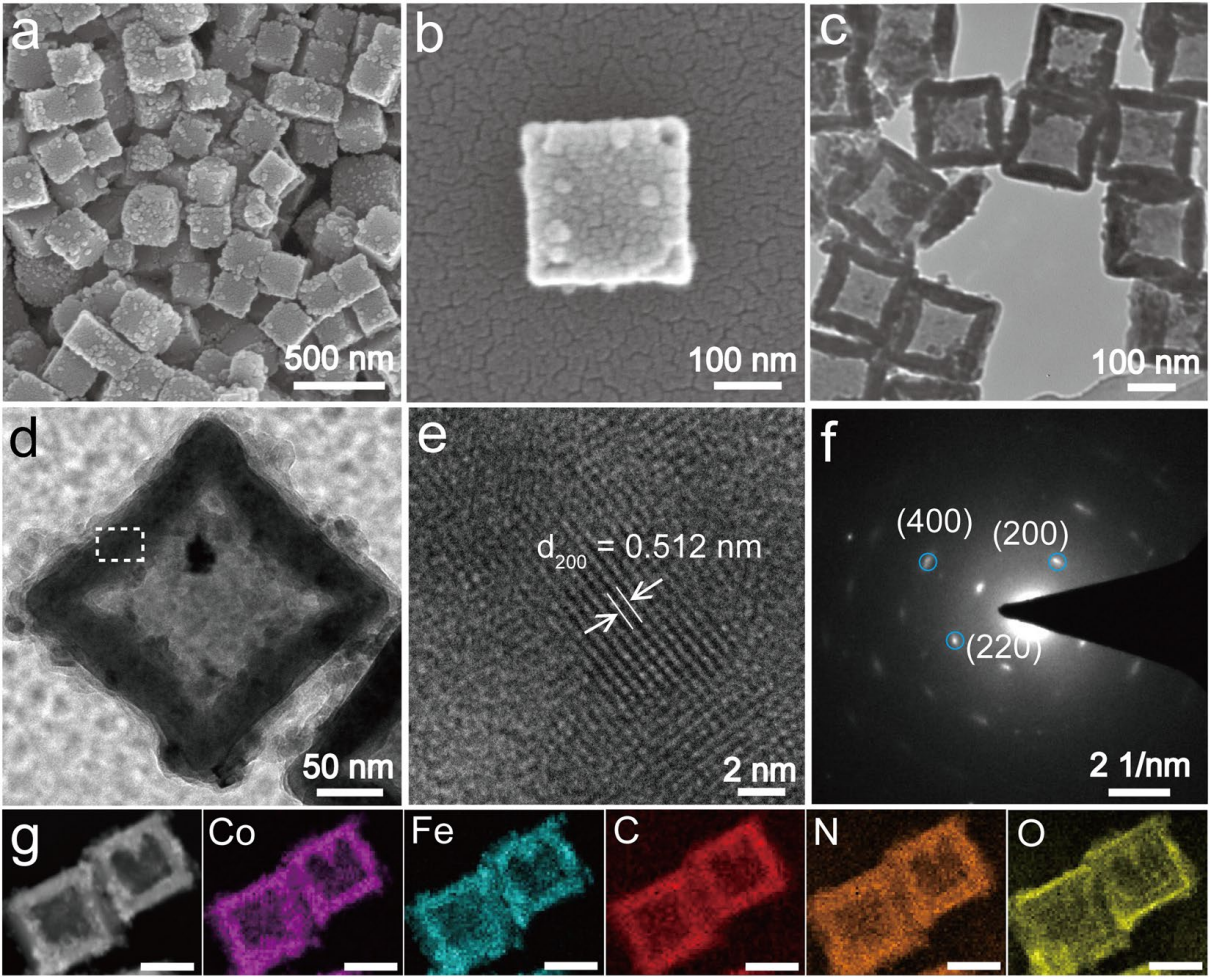
(**a**,**b**) SEM and (**c**,**d**) TEM of the as-synthesized CFHC. (**e**) HRTEM of the rectangular area in panel d showing a (200) crystalline plane distance of 0.512 nm. (**f**) SAED pattern. (**g**) HAADF-TEM image and the corresponding elemental mapping for Co (purple), Fe (cyanide blue), C (red), N (orange) and O (yellow), scale bar 100 nm.

The CFHC was obtained by solvothermal treatment of the initial solid CFC particles (Supplementary Fig. S1) under the protection of PVP. As can be seen from the SEM (Fig. 1a,b) and TEM (Fig. 1c,d) images, the as-synthesized CFHC displayed a 3D hollow cubic shape with a particle size of approximately 200 nm. The surface of the cage shell become rough consisting of Co-Fe PBA nanoparticles and the thickness of the shell was around 30 nm as revealed in Fig. 1d. Notably, the hollow architectures were truncated at the eight corners during the solvothermal process, leading to an open framework (Fig. 1a,b, Supplementary Figs S2 and S3). High-resolution TEM (HRTEM) acquired from the individual cage marked in Fig. 1d demonstrated a crystal lattice fringe spacing of 0.512 nm corresponding to the (200) plane as evidenced in Fig. 1e, which was in good agreement with the typical face-centered cubic (fcc) PBA crystal phase^45^. A distinct selected area electron diffraction (SAED) pattern (Fig. 1f) showed clear diffraction spots corresponding to the crystal planes of (200), (220) and (400), indicative of a high-quality single-crystal structure of CFHC^39^. Besides, the Co, Fe, C, N and O elemental mapping in CFHC was depicted in Fig. 1g. It was worth noting that a combination analysis of the high-angle annular dark field TEM (HAADF-TEM) image and the elemental mapping suggested that the N and O were mainly distributed on the inner and outer surface of CFHC, which could be ascribed to the adsorption of the hydrophilic PVP molecules on the cage (Fig. 1d and Supplementary Fig. S4) during the solvothermal reaction. Moreover, thermogravimetry analysis (TGA) measurement of CFHC illustrated a larger weight loss in the range from 150 to 800 °C compared with CFC (Supplementary Fig. S5), which fell into the region of deformation of PVP molecules and PBA frameworks, further evidencing the existence of PVP on the surface of CFHC. Fascinatingly, further experiment demonstrated that this synthesis strategy was also applicable to obtain NFHC using NFC as the precursor (Supplementary Fig. S6).

**Figure 2:**
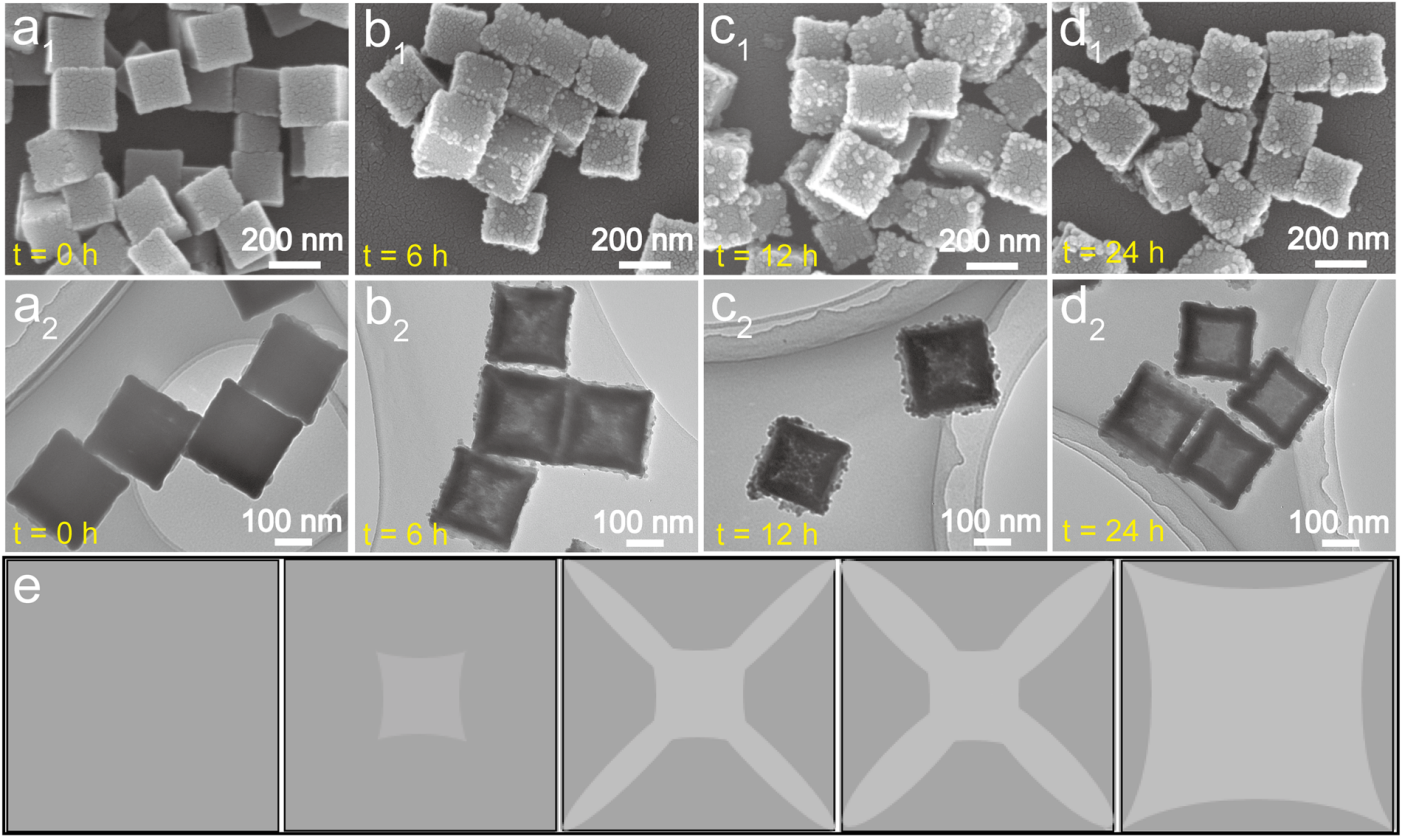
Time-dependent SEM (panel 1 of a–d) and TEM (panel 2 of a–d) images of CFHC at different reaction time, (**a**) 0 h, (**b**) 6 h, (**c**) 12 h and (**d**) 24 h. (**e**) Schematic illustration showing the structure evolution of CFHC.

Time-dependent experiment was then carried out to track the structure evolution from solid cubes to 3D hollow open cages. The morphology and structure of several intermediate samples of CFC collected at different reaction stages were displayed in Fig. 2 and Supplementary Fig. S7. After 1 h, the inner center part of the initial CFC started to dissolve. As the reaction time went by, the center cavity become extended preferentially along the diagonal of the cubes. After 24 h, the interior moiety of the cubes completely disappeared, and consequently a hollow structure was formed. A schematic illustration of structure evolution proceeding was displayed in Fig. 2e. Time-dependent XRD patterns (Supplementary Fig. S8) suggested that all the intermediate products inherited the fcc crystalline structure from their parent CFC with a chemical composition of Co_3_[Fe(CN)_6_]·10H_2_O (JCPDS 00-046-0907)^50^.

**Figure 3:**
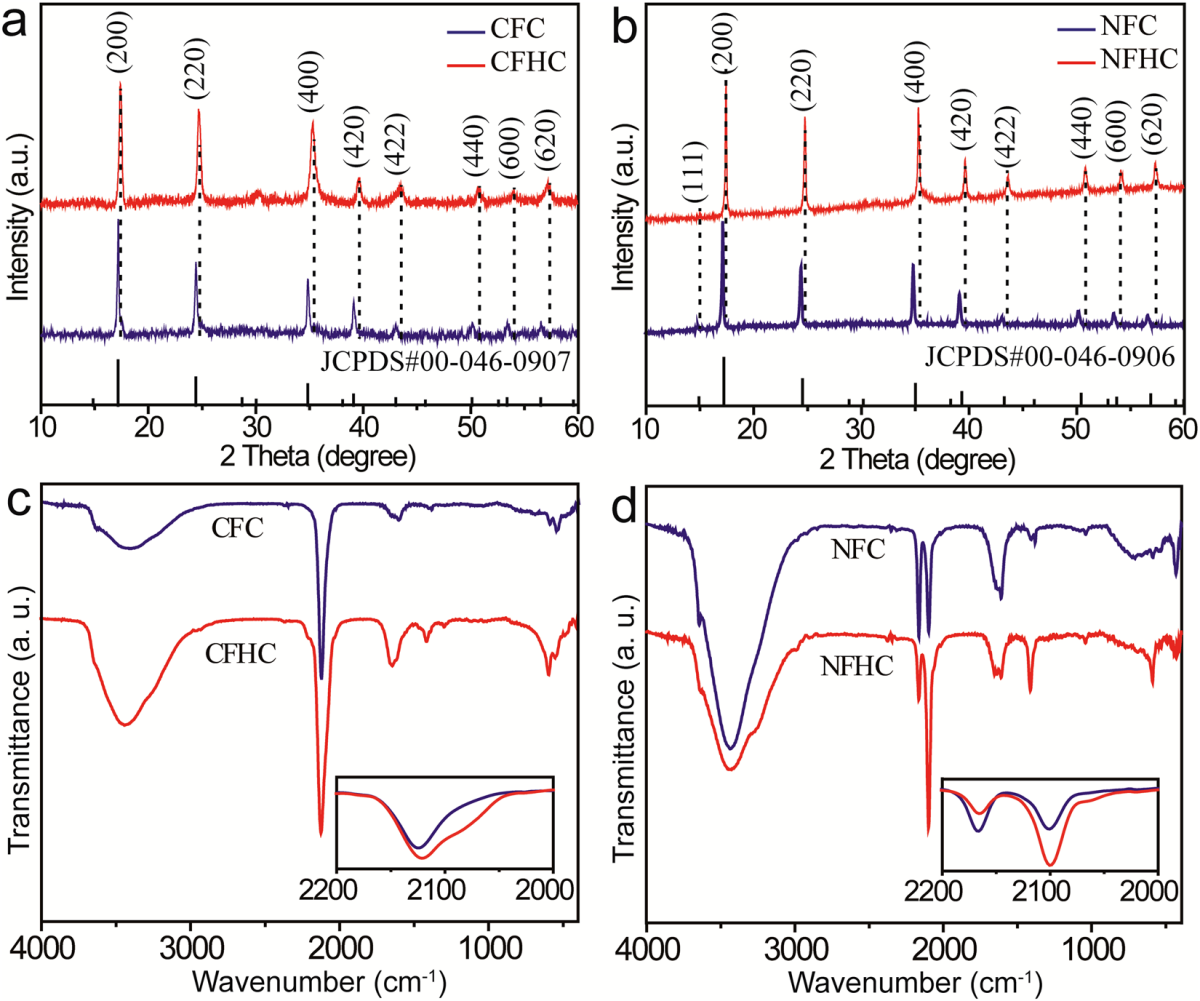
(**a**,**b**) XRD patterns of CFC (blue), CFHC (red), NFC (blue) and NFHC (red), respectively. The dashed lines indicated the peak shift of CFHC and NFHC relative to CFC and NFC, respectively. (**c**,**d**) FTIR spectra of CFC (blue), CFHC (red), NFC (blue) and NFHC (red), respectively, insets showing the enlarged spectra ranging from 2000 to 2200 cm^−1^.

Further analysis of the XRD patterns of CFHC and NFHC revealed an appealing structure transformation. As can be seen from Fig. 3a,b, the XRD peaks of both CFHC and NFHC upshifted progressively relative to their pristine CFC and NFC counterpart, respectively, indicating a cell parameter contraction from 10.295 Å (CFC) and 10.229 Å (NFC) to 10.269 Å (CFHC) and 10.208 Å (NFHC), respectively, due to the structural transformation from Ni^П^-CN-Fe^Ш^ to Co^Ш^-CN-Fe^П^ ^51–54^. Such a structure contraction accompanied with a charge transfer process was further evidenced by the FTIR spectra shown in Fig. 3c,d. Compared with CFC, the υ (CN) vibrational peak for Co^Ш^-CN-Fe^П^ (2122 cm^−1^) in CFHC was enhanced and a new peak on the shoulder at 2078 cm^−1^ corresponding to Co^П^-CN-Fe^П^ was observed^40,45,51,53^. Similarly in NFHC, the υ (CN) vibrational peak for Ni^Ш^-CN-Fe^П^ (2113 cm^−1^) increased significantly while the peak belong to Ni^П^-CN-Fe^Ш^ (2158 cm^−1^) decreased^53^. Such charge transfer from M^П^ in the initial PBA cubes to M^Ш^ in the hollow cages (M = Co or Ni) would facilitate the electrocatalytic oxidation process. In addition, the peaks located at 1412 and 1640 cm^−1^ for CFHC and 1414 and 1657 cm^−1^ for NFHC were ascribed to bending vibration of –CH_2_– and stretching vibration of –C=O– (Supplementary Fig. S9), respectively, confirming the presence of PVP moiety on the surface of CFHC and NFHC.

**Figure 4:**
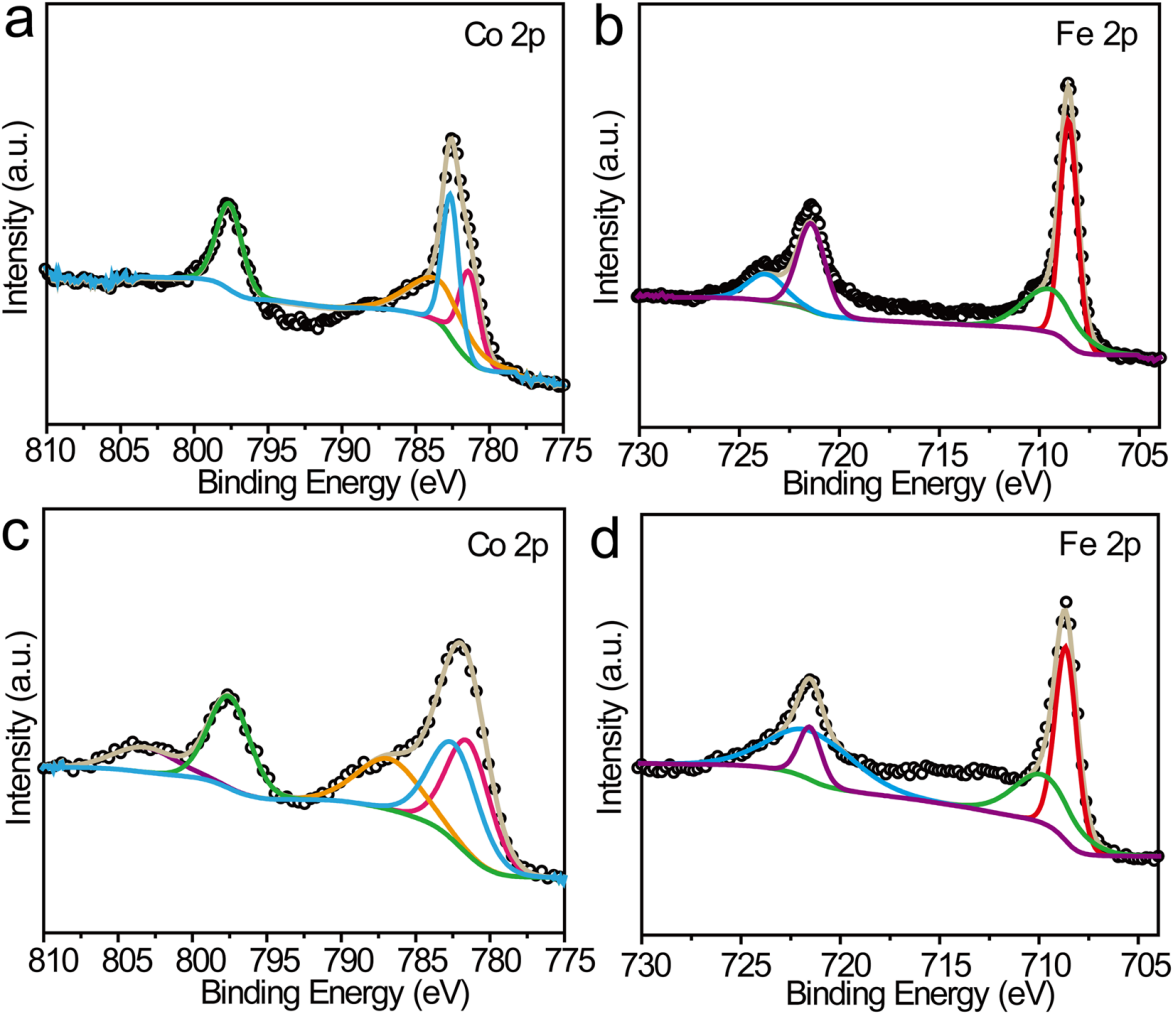
XPS spectra of Co 2p (**a**,**c**), Fe 2p (**b**,**d**) for CFC (**a**,**b**) and CFHC (**c**,**d**).

The valence state of the 3D hollow cubic cages was further investigated by XPS. As displayed in Fig. 4a,c, the dominant peaks located at 781.4 and 797.6 eV were ascribed to Co^3+^ 2p_3/2_ and 2p_1/2_, respectively, and the binding energy at 782.6 eV was assigned to Co^2+^ 2p_3/2_^24,39,42,50^. It was obvious that in CFHC the XPS peaks at 781.4 eV for Co^3+^ were enhanced while the peak corresponding to Co^2+^ at 782.6 eV was decreased (Fig. 4c). Synchronously, the XPS peak for Fe^3+^ located at 708.5 eV was decreased and the peak for Fe^2+^ at 721.4 eV was increased (Fig. 4b,d)^39,42,50^. Such an increase of M^Ш^/M^П^ peak ratio was also observed for NFHC as shown in Supplementary Figs S10 and S11. This valence alteration can be interpreted by the electron transfer from the anti-bonding e_g_^*^ orbital of M^П^ to the bonding t_2g_ orbital of M^Ш^ in order to maximize the ligand field stabilization energy^31^. It can be inferred that the solvothermal treatment triggered such electron transfer process.

### The possible mechanism for the formation of CFHC and NFHC

In order to unveil the driving force for the formation of the hollow cages and the reason for the electron transfer in CFHC and NFHC, the solvothermal process for the formation of CFHC as an example was systematically investigated. It was found that the reaction temperature and the PVP played a critical role in constructing the hollow cubic open frameworks. When the reaction temperature was decreased to 160 °C, it needed longer time to form the hollow cage (Supplementary Fig. S12a,b). Whereas if the temperature was increased to 200 °C, the obtained cage was collapsed into pieces and some very thin nanosheets were observed (Supplementary Fig. S12c,d). On the other hand, if the reaction was carried out without addition of PVP, the as-obtained hollow cage become fragile featuring a shell thickness of merely 10 nm (Supplementary Fig. S13).

Considering the ionizing of EtOH as Eq. (1):

1 $${{\rm{CH}}}_{3}{{\rm{CH}}}_{2}{\rm{OH}}\to {{\rm{CH}}}_{3}{{\rm{CH}}}_{2}{{\rm{O}}}^{-}+{{\rm{H}}}^{+}$$

Although the pK_a_ value of EtOH is rather large (15.9 at 25 °C)^55^, the ionization equilibrium under solvothermal reaction ensured the sufficient proton in the reaction system. Experimentally, the pH values before and after the solvothermal reaction were measured to be 8.12 and 11.20, respectively, indicating the depletion of proton in the system. “Accordingly, it can be deduced that the hollow” cage architectures was probably formed by the etching effect of proton released from EtOH during the solvothermal proceeding^56,57^. This speculation was derived from the fact that similar hollow structures can be synthesized by using acid (HCl) as etching reagents^48,49^. The *in-situ* released protons from EtOH acting as a very weak acid were able to diffuse into the interior of CFC along its nanoporous channels driven by the concentration gradient. When the local proton concentration in the center part of CFC was large enough to break the bond of CFC, the etching started to extend along the high-energy body diagonal of CFC to the corners progressively. During this process, the structure of CFC shrank in order to stabilize the framework. Simultaneously, the charge transfer process occurred as following:2

In this proceeding, PVP served as a protection shell to slow down the etching speed which can easily absorb on the surface of the initial cubes due to the strong coordination ability of iron ions in CFC to the amide moiety in PVP^46,49^.

**Figure 5:**
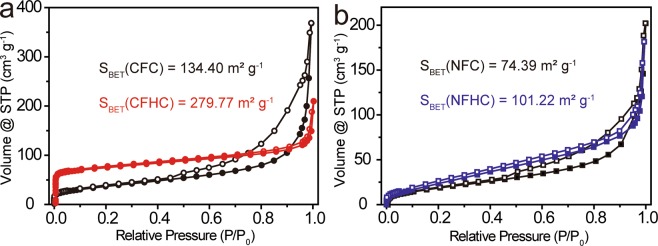
Nitrogen gas adsorption (solid) and desorption (empty) isothermal curves of (**a**) CFC (black) and CFHC (red), and (**b**) NFC (black) and NFHC (blue).

The porous hollow cavity of CFHC and NFHC were then investigated by nitrogen gas adsorption/desorption isotherms. As illustrated in Fig. 5, the Brunauer-Emmett-Teller (BET) specific surface area of CFHC and NFHC were measured to be 279.77 and 101.22 m^2^ g^−1^, respectively, both of which were extensively larger than their corresponding initial counterparts. The increased specific surface area could enable more active catalytic sites exposed, thus facilitating the electrocatalytic OER and UOR process.

### Electrocatalytic performance of CFHC and NFHC as OER catalyst

**Figure 6:**
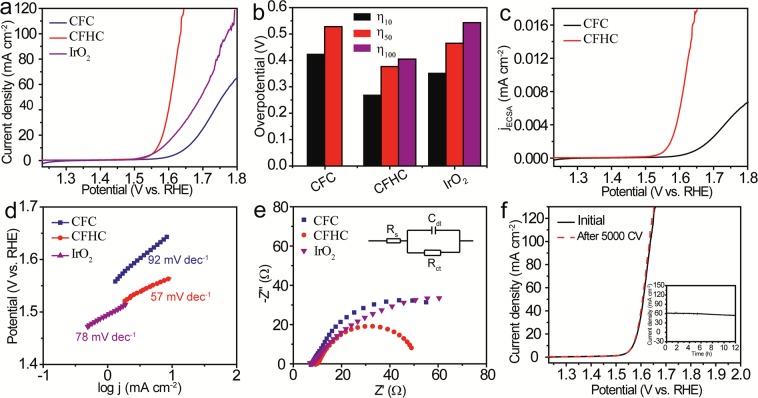
OER performance of the CFHC catalyst. (**a**) LSV curves of CFC, CFHC and IrO_2_ and (**b**) their corresponding overpotentials showing η_10_, η_50_, η_100_ measured in 1 M KOH with a scan rate of 5 mV s^−1^. (**c**) ECSA-normalized LSV curves of CFC and CFHC. (**d**) Tafel slopes and (**e**) EIS of CFC, CFHC and IrO_2_, inset in e showing the equivalent circuit diagram. (**f**) LSV curves of CFHC before (solid black) and after (dashed red) 5000 CV cycles, inset showing the chronoamperometic i-t curve measured at overpotentail of 380 mV.

To demonstrate the enhanced electrocatalytic performance of the newly-synthesized 3D hollow architectures, the OER experiments were firstly conducted under a traditional three-electrode setup taking CHFC as an example. Figure 6a depicted the linear scanning voltammetry (LSV) curves of CFHC measured in 1 M KOH aqueous solution with a scan rate of 5 mV s^−1^. For comparison, CFC and the commercial benchmark IrO_2_ were also examined as references under the same condition. Apparently, compared to the latter two, the polarization curve of CFHC exhibited a dramatic superiority with an overpotential of merely 330 mV at a current density of 10 mA cm^−2^ (η_10_), much smaller than those of CFC (423 mV) and IrO_2_ (351 mV). Even at larger current density, CFHC still displayed outstanding performance, i. e., the η_50_ and η_100_ of CFHC were the smallest among three tested samples (Fig. 6b). The electrochemical active surface area (ECSA) of the catalyst was reflected from the electric double-layer capacitance (C_dl_), which can be obtained by cyclic voltammetry (CV) scanning in a non-Faradic potential range^9^. The C_dl_ of CFHC (390 mF cm^−2^) was larger than that of CFC (282 mF cm^−2^) (Supplementary Fig. S14), which indicated that CFHC owned a larger ECSA, contributing to a superior electrocatalytic activity. To be more convinced, the current density was normalized by ECSA in order to obtain the intrinsic polarization curves^58^. As shown in Fig. 6c, in this case the CFHC catalyst can maintain its domination. Furthermore, the Tafel plots was derived from the polarization curves following the equation of η = *a* + *b* log j (*b* is Tafel slope, *a* is a constant)^43^. From Fig. 6d, it can be seen that the Tafel slope of the CFHC catalyst (57 mV dec^−1^) was smaller than those of CFC (92 mV dec^−1^) and IrO_2_ (78 mV dec^−1^), indicative of a faster catalytic kinetics. The electrochemical impedance spectroscopy (EIS) in Fig. 6e suggested that CFHC exhibited a smaller semicircle diameter than CFC and IrO_2_, revealing a favorable charge transfer resistance (R_ct_) of CFHC (38.4 Ω) compared with CFC (58.4 Ω) and IrO_2_ (52.4 Ω)^9^. The above result demonstrated that the CFHC was a remarkable catalyst that outperformed the commercial IrO_2_ and most of the recently reported non-precious metal catalysts in alkaline (Supplementary Table S1). The stability of the CFHC catalyst was evaluated by continuous scanning of the CV curves. Notably, after 5000 CV cycles, the LSV curve was almost identical with the initial one (Fig. 6f). Besides, the long-term chronoamperometry test (i-t curve in the inset of Fig. 6f) manifested that the electrocatalytic activity can retain at least for 12 h. The OER activity of NFHC was then accessed as shown in Supplementary Figs S15 and S16, which also displayed desirable catalytic properties. The morphology of the CFHC and NFHC catalyst could preserved after the long-term chronoamperometic measurement (Supplementary Figs S17 and S18) further confirmed their durability in alkaline media.

### Electrocatalytic performance of CFHC and NFHC as UOR catalyst

**Figure 7:**
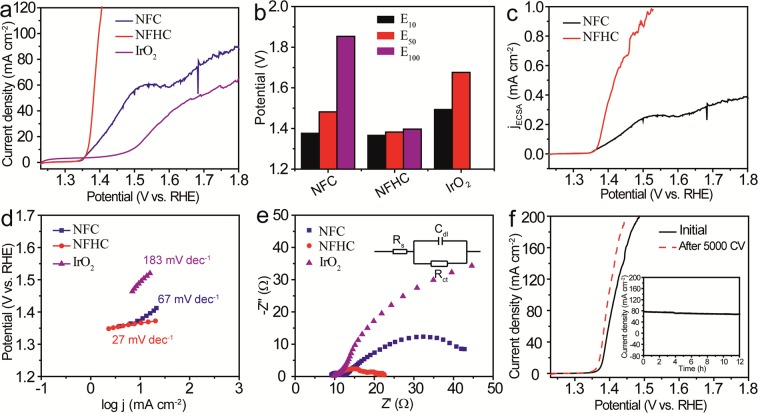
UOR performance of the NFHC catalyst. (**a**) LSV curves of NFC, NFHC and IrO_2_ and (**b**) their corresponding potentials acquired at E_10_, E_50_, E_100_ measured in 1 M KOH containing 0.5 M urea with a scan rate of 5 mV s^−1^. (**c**) ECSA-normalized LSV curves of NFC and NFHC. (**d**) Tafel slopes and (**e**) EIS of NFC, NFHC and IrO_2_, inset in e showing the equivalent circuit diagram. (**f**) LSV curves of NFHC before (solid black) and after (dashed red) 5000 CV cycles, inset showing the chronoamperometic i-t curve measured with potential of 1.39 V.

Furthermore, the UOR performance of the NFHC and CFHC catalysts were subsequently evaluated. As shown in Fig. 7a, the polarization curve of NFHC measured in 1 M KOH containing 0.5 M urea aqueous solution displayed an improved UOR performance compared with NFC and IrO_2_. To deliver a current density of 10 mA cm^−2^, the NFHC catalyst required a potential (E_10_) of 1.37 V, lower than those of NFC (1.38 V) and IrO_2_ (1.49 V). More impressively, the NFHC catalyst only needed 1.40 V to obtain a current density of 100 mA cm^−2^, decreased by 24.32% compared with NFC at the same lever (Fig. 7b). The C_dl_ value of NFHC (12.1 mF cm^−2^) was larger than that of its counterpart NFC (9.3 mF cm^−2^) (Supplementary Fig. S19), demonstrating a favorable electrocatalytic activity of NFHC. The ECSA-normalized LSV curve shown in Fig. 7c confirmed the superior UOR behavior of NFHC. The Tafel slopes of NFHC, NFC and IrO_2_ were 27, 67 and 183 mV dec^−1^ successively (Fig. 7d), indicating a dramatically faster catalytic kinetics of NFHC during the UOR process. Moreover, the R_ct_ value of NFHC was 8.3 Ω, much smaller than those of NFC (26.5 Ω) and IrO_2_ (40.1 Ω) (Fig. 7e), implying that the NFHC catalyst underwent a favorable charge transfer process. Notably, the UOR performance of the NFHC catalyst was superior to the commercial IrO_2_ and comparable to most previously reported non-precious metal-based catalysts (Supplementary Table S2). In addition, the LSV curve after continuous CV scanning and long-term i-t test were performed as shown in Fig. 7f. It was clear that after 5000 CV cycles, the LSV curve of the NFHC catalyst exhibited negligible change and the electrocatalytic activity can preserve at least for 12 h, indicative of a desirable catalytic stability. Moreover, the UOR performance of CFHC was also evaluated as shown in Supplementary Figs S20–S22, and the result confirmed the enhancement of oxidative catalytic properties of CFHC relative to the solid CFC precursor. Moreover, the morphology of both CFHC and NFHC could maintained after the long-term i-t test (Supplementary Figs S23 and S24), indicative of robust catalytic stability of these novel electrocatalysts.

## Discussion

The above results demonstrated that the CFHC catalyst was a desirable efficient bifunctional electrocatalyst for OER and UOR. The outstanding electrocatalytic performance of CFHC could be attributed to the following reasons: (i) the 3D hollow open framework of CFHC provided larger BET specific surface area and more electrochemical active surface area exposed to the electrolyte, which could extremely facilitate the mass transportation during the reaction, hence increasing the catalytic reaction kinetics of the OER and UOR process^16,48^. (ii) The *in-situ* formed oxidative species of Co^Ш^-CN-Fe^П^ state induced by the electron transfer process during the solvothermal reaction was highly reactive towards OER and UOR, which definitely accelerated the charge transfer process during the water and urea oxidation reaction^59^. Therefore, taking the advantages of mass and charge transportation to the electrolysis, a dramatic improvement of the OER and UOR performance of the CFHC catalyst was displayed.

In summary, a self-templated 3D hollow cubic open frameworks, CFHC and NFHC, were constructed through a facile solvothermal treatment of the solid precursors, which can be directly utilized as water and urea oxidation catalysts. It was inferred that the proton released from the alcohol solvent induced the formation of the hollow architectures by etching effect, accompanied with a charge transfer process. The as-obtained CFHC and NFHC with larger specific geometrical and electrochemical surface area exhibited a dramatically improved OER and UOR performance, which showed lower oxidation potential, faster catalytic kinetics and favorable durability, demonstrating that CFHC and NFHC were highly efficient bifunctional electrocatalyst. Impressively, the CFHC catalyst exhibited an overpotential of 330 mV to deliver a current density of 10 mA cm^−2^ in the OER process, and NFHC required a potential of only 1.40 V to drive a current density of 100 mA cm^−2^ for urea electrolysis, decreased by 21.99% and 24.32% compared to their counterparts CHC and NHC, respectively, at the same lever. Our work make a new contribution to the design and construction of intricate PBA-based architectures in the application of electrochemical energy conversion, and would motivate more extensive research on other fields.

## Materials and Methods

### Synthesis of CFC and NFC

Co-Fe cubes (CFC) and Ni-Fe cubes (NFC) were prepared via the precipitation method according to the previous literatures^50,60^. Briefly, CoCl_2_∙6H_2_O (0.6 mmol) and TCD (0.9 mmol) were dissolved in 20 mL of DI water to form a transparent solution, which was then poured into a solution of K_3_[Fe(CN)_6_] (2 mM) under vigorous stirring. After several minutes, the mixtures were left to stand for 20 h without any disturbance. The thus-obtained CFC were collected by centrifugation at 8500 rpm for 15 min, followed by washing with water and EtOH three times, and then dried at 70 °C for 12 h. NFC were synthesized via a similar procedure except Ni(NO_3_)_2_∙6H_2_O was used instead of CoCl_2_∙6H_2_O.

### Synthesis of CFHC and NFHC

The Co-Fe and Ni-Fe PBA hollow cage, designed as CFHC and NFHC respectively, were synthesized through a solvothermal treatment. Experimentally, 20 mg of the as-synthesized CFC/NFC were dispersed in 20 mL of EtOH with the assistance of ultrasonication. This dispersion was then added into 20 mL of EtOH solution containing 100 mg of PVP under vigorously stirring. The obtained transparent solution was transferred to a Teflon-lined autoclave capped with a stainless steel vessel. After heating at 180 °C for 24 h, the autoclave was cooled naturally to room temperature. The precipitates were collected by centrifuging at 8500 rpm for 15 min and washed with DI water and EtOH three times and dried at 70 °C for 12 h.

### Electrocatalytic performance characterization

All the electrochemical measurements were performed on a CHI660E (Chenhua, Shanghai) electrochemical workstation with the standard three-electrode system. A glassy carbon electrode (GCE, Φ = 3 mm), a graphite rod and a saturated calomel electrode (SCE) were used as working electrode, counter electrode and reference electrode, respectively. The working electrode was prepared as follow: firstly 10 mg of catalyst powder and 5 mg of black carbon were dispersed in 500 μL of IPA/DI water (v/v = 4:1) under ultrasonication to form a homogenous ink, then ca. 2 μL of the ink was drop-casted on the surface of GCE followed by coating with 2 μL of Nafion solution and dried in air. OER performance were conducted in 1 M KOH aqueous solution and UOR in 1 M KOH containing 0.5 M urea aqueous solution. The obtained polarization curves were calibrated with iR loss using the equation of *E*_cal._ = *E* − iR. All the potentials were converted to the reversible hydrogen electrode (RHE) following the equation of *E*_RHE_ = *E*_SCE_ + 0.24 + 0.059 pH^41^. Electrochemical impedance spectroscopy (EIS) measurements were recorded under open circuit potentials with the frequency ranging from 0.1 Hz to 100 kHz. For the double-layer capacitor (C_dl_) data, cyclic voltammetry (CV) curves were recorded in the non-Faradic region with scanning rate of 2, 4, 6, 8, 10 and 12 mV s^−1^, and the C_dl_ can be obtained by plotting the current difference (Δj) against the scanning rate.Electrochemical active surface area (ECSA) was estimated by the equation:

$${\rm{ECSA}}={{\rm{C}}}_{{\rm{dl}}}/{{\rm{C}}}_{{\rm{s}}}\times {\rm{S}}$$

where C_s_ refers to the specific capacitance on the electrode surface and S is the actual area of the working electrode.Generally, C_s_ is in the range of 20–60 μF cm^−2^, herein for the CFHC catalyst, the averaged C_s_ value of 40 μF cm^−2^ was used according the literatures^61,62^.

## Supplementary information

Suporting Information
