# Supplementary material for: Boosting the activity of Prussian-blue analogue as efficient electrocatalyst for water and urea oxidation
Source: Sci Rep. 2019 Nov 4;9:15965. doi: 10.1038/s41598-019-52412-1 (PMC6828720; doi:10.1038/s41598-019-52412-1)
Supplement: Supplementary file 1 — Suporting Information [file 41598_2019_52412_MOESM1_ESM.pdf]

Supplementary Information

Boosting the activity of Prussian-blue analogue as efficient electrocatalyst for water and urea oxidation

Yongqiang Feng,[1,*] *Xiao Wang,*[1] *Peipei Dong,*[1] *Jie Li,*[2] *Li Feng,*[1] *Jianfeng Huang,*[1,*] *Liyun Cao,*[1] *Liangliang Feng,*[1] *Koji Kajiyoshi,*[3] *Chunru Wang*[2,*]

[1]School of Materials Science and Engineering, Shaanxi Key Laboratory of Green Preparation and Functionalization for Inorganic Materials, Key Laboratory of Auxiliary Chemistry and Technology for Chemical Industry, Ministry of Education, Shaanxi University of Science and Technology, Xi'an, 710021, People's Republic of China

[2]Beijing National Laboratory for Molecular Sciences, Laboratory of Molecular Nanostructure and Nanotechnology, Institute of Chemistry, Chinese Academy of Sciences, Beijing 100190, China

[3]Research Laboratory of Hydrothermal Chemistry, Faculty of Science and Technology, Kochi University, Kochi, 780-8520, Japan

Contents

Figure S1. (a) SEM, (b, c) TEM and (d) HRTEM of CFC. 4

Figure S2. SEM image of CFHC. 5

Figure S3. SEM image of CFHC. 6

Figure S4.TEM image of CFHC. 6

Figure S5. TGA pattern of CFHC (red) and CFC (black). 7

Figure S6. (a) SEM and (b) TEM images of NFC. (c) SEM and (d) TEM images of NFHC. (e) HRTEM and (f) SAED pattern of NFHC. (g) HAADF-TEM image of NFHC and the corresponding elemental mapping for Co (cyanide blue), Fe (purple), C (red), N (orange) and O (yellow), scale bar 100 nm. 9

Figure S7. Time-dependent SEM (panel 1 of a-e) and TEM (panel 2 of a-e) images of CFHC at different reaction time, (a) 0 h, (b) 1 h, (c) 6 h and (d) 12 h, and (e) 24 h. 10

Figure S8. Time-dependent XRD patterns of CFHC at different reaction time. The dashed lines indicated the peak shift of CFHC relative to CFC. 10

Figure S9. FTIR spectra of (a) CFC (black) and CFHC (red), and (b) NFC (black) and NFHC (red), respectively, in the range from 1200 to 1800 cm^{-1} 11

Figure S10. Overlay XPS spectra of CFHC (a) and NFHC (b). 12

Figure S11. XPS spectra of Ni 2p (a ,c), Fe 2p (b, d) for NFC (a, b) and NFHC (c, d). 12

Figure S12. (a) SEM and (b) TEM images of CFHC, reaction condition: 100 mg PVP, 160 ℃, 24 h.(c) SEM and (d) TEM images of CFHC, reaction condition: 100 mg PVP, 200 ℃, 24 h. 13

Figure S13. (a) SEM and (b) TEM images of CFHC, reaction condition: 0 mg PVP, 180 ℃, 24 h. 14

Figure S14. CV curves of (a) CFC and (b) CFHC with different scan rate from 2 to 12 mV in the range of 0.04-0.16 V in 1 M KOH, and (c) the corresponding current density difference at 0.10 V plot against the scan rate. 15

Figure S15. OER performance of the NFHC catalyst. (a) LSV curves of NFC, NFHC and IrO_2 and (b) their corresponding overpotentials showing η_{20}, η_{50}, η_{100} measured in 1 M KOH with a scan rate of 5 mV s^{-1}. (c) Tafel slopes and (d) EIS of NFC, NFHC and IrO_2, inset in d showing the equivalent circuit diagram. (e) LSV curves of NFHC before (solid black) and after (dashed red) 5000 CV cycles and (f) showing the i-t curve measured at 1.58 V. 15

Figure S16. CV curves of (a) NFC and (b) NFHC with different scan rate from 2 to 12 mV in the range of 0.04-0.16 V in 1 M KOH, and (c) the corresponding current density difference at 0.10 V plot against the scan rate. 16

Figure S17. SEM image of CFHC after i-t measurement during the OER process.................... 16

Figure S18. SEM image of NFHC after i-t measurement during the OER process. 17

Figure S19. CV curves of (a) NFC and (b) NFHC with different scan rate from 2 to 10 mV in the range of 0.04-0.16 V in 1 M KOH containing 0.5 M urea, and (c) the corresponding current density difference at 0.10 V plot against the scan rate. 18

Figure S20. UOR performance of the CFHC catalyst. (a) LSV curves of CFC, CFHC and IrO_2 and (b) their corresponding Tafel slopesmeasured in 1 M KOH containing 0.5 M urea solution with a scan rate of 5 mV s^{-1}. (c) EIS of CFC, CFHC and IrO_2, inset in c showing the equivalent circuit diagram. (d) LSV curves of CFHC before (solid black) and after (dashed red) 5000 CV cycles, inset showing the i-t curve measured at 1.46 V. 18

Figure S21. CV curves of (a) CFC and (b) CFHC with different scan rate from 2 to 12 mV in the range of 0.04-0.16 V in 1 M KOH containing 0.5 M urea, and (c) the corresponding current density difference at 0.10 V plot against the scan rate. 19

Figure S22. ECSA-normalized LSV curves of CFC and CFHC during UOR process. 19

Figure S23. SEM image of CFHC after i-t measurement during the UOR process. 20

Figure S24. SEM image of NFHC after i-t measurement during the UOR process. 20

Table S1. The OER properties of CFHCand NFHC compared with other non-precious metal-based OER catalysts in 1 M KOH. 21

Table S2. The UOR properties of CFHCand NFHC compared with other non-precious metal-based UOR catalysts in 1 M KOH with urea. 22

1. Materials and instrumentation

Potassium hexacyanoferrate(III) ($K_3[Fe(CN)_6]$, ⩾ 98%), cobalt(II) chloride hexahydrate ($CoCl_2·6H_2O$, ⩾ 98%), trisodium citrate dihydrate (TCD, $C_6H_5Na_3O_7·2H_2O$, ⩾ 99%), nickel(II) nitrate hexahydrate ($Ni(NO_3)_2·6H_2O$, ⩾ 98%), polyvinylpyrrolidone (PVP, $(C_6H_9NO)_n$, K25), ethanol (EtOH, 98%) were purchased from FUJIFILM Wako Pure Chemical Corporation. Urea ($CO(NH_2)_2$, ⩾ 99%) were purchased from Sinopharm Chemical Reagent Co., Ltd., potassium hydroxide (KOH, ⩾ 85%) and isopropyl alcohol (IPA, $(CH_3)_2CHOH$, ⩾ 98%) were received from Kermel, Nafion solution (5%) and IrO_2 (99.9%) were purchased from Sigma-Aldrich and Energy Chemical, respectively, and deionized (DI) water (resistivity: 18.3 MΩ·cm) were produced by an ultrapure water system (ULUPURE, UPDR-I-10T). All the chemical reagents were used as received without further treatment.

Field-emission scanning electron microscopy (FE-SEM) was performed on a JEOL S4800 instrument. Transmission electron microscopy (TEM) images and element mapping analysis were conducted on a FEI Tecnai G2 F20 S-TWIN instrument. Powder X-ray diffraction (XRD) were exerted on a Rigaku D/max-2200PC diffractometer (Japan) with Cu Kα radiation (λ = 0.15418 nm). Fourier transform infrared spectroscopy (FTIR) was carried out on a Bruker Vector-22 machine. X-ray photoelectron spectroscopy (XPS) spectra were collected on the Thermo Scientific ESCALab 250Xi using 200 W monochromated Al Kα radiation. Nitrogen adsorption/desorption isotherm curves were recorded on a Quantachrome Autosorb AS-1 instrument at 77 K. Electrochemical measurements were conducted on the CHI660E (Chenhua, Shanghai) instrument.

2. Synthesis and characterization of CFC

CFC was synthesized through a precipitation method according to the previous literature.[1] Briefly, $CoCl_2·6H_2O$ (0.6 mmol) and TCD (0.9 mmol) were dissolved in 20 mL of DI water to

form solution A, and of $K_3[Fe(CN)_6]$ (0.4 mmol) was added to another 20 mL of DI water to form solution B. Then solution A were poured into solution B under vigorous stirring. After several minutes, the mixtures were left to stand for 20 h without any disturbance. The thus-obtained CFC were collected by centrifugation at 8500 rpm for 15 min, followed by washing with water and EtOH three times, and then dried at 70 °C for 12 h. Figure S1 showed the SEM and TEM images of the as-synthesized CFC. As can be seen, CFC exhibited a solid cubic morphology with an average size of 200 nm, the HRTEM image in Fig. S1d revealed a distinct fringe crystal spacing of 0.518 nm, corresponding to the (200) plane of the cubic PBA with a space group of $F\bar{4}3m$ (216).[2]

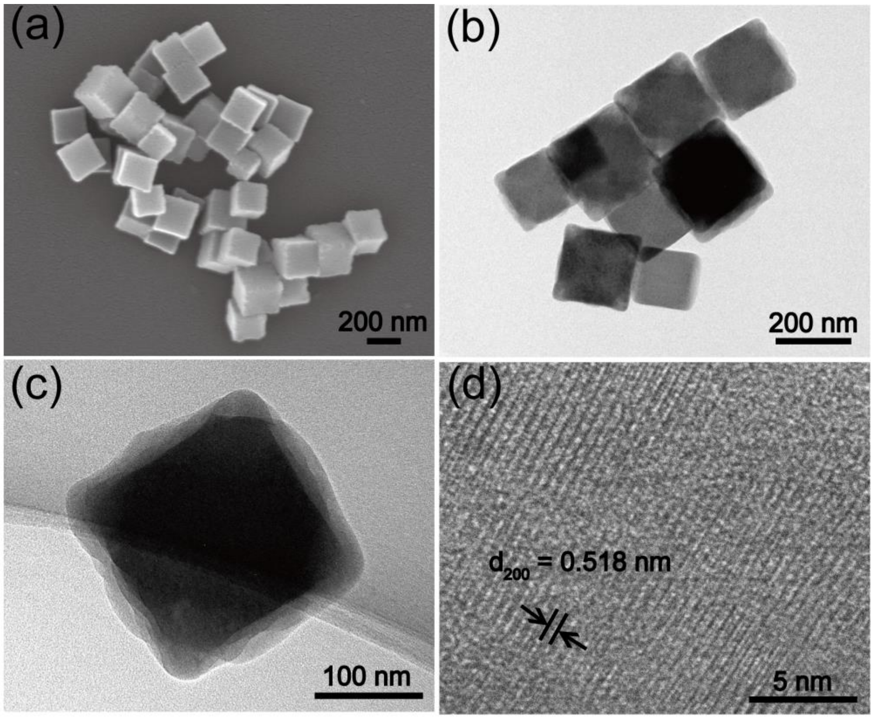

Figure S1. (a) SEM, (b, c) TEM and (d) HRTEM of CFC.

3. Synthesis and characterization of CFHC

The CFHC was synthesized through a solvothermal treatment. Experimentally, 20 mg of the as-synthesized CFC were dispersed in 20 mL of EtOH with the assistance of ultrasonication. This dispersion was then added into 20 mL of EtOH solution containing 100 mg of PVP under vigorously stirring. The obtained transparent solution was transferred to a Teflon-lined autoclave capped with a stainless steel vessel. After heating at 180 ℃ for 24 h, the autoclave was cooled naturally to room temperature. The precipitates were collected by centrifuging at 8500 rpm for 15 min and washed with DI water and EtOH three times and dried at 70 ℃ for 12 h. The morphology of the as-synthesized CFHC was shown in Fig. S2 and S3. From the HRTEM image shown in Fig. S4, it was apparent that on the inner and outer surface there existed a thin layer of polymer, which was ascribed to the adsorbed PVP molecules. The hydrophilic PVP could promote the adsorption of water and urea, thus facilitating the OER and UOR process.

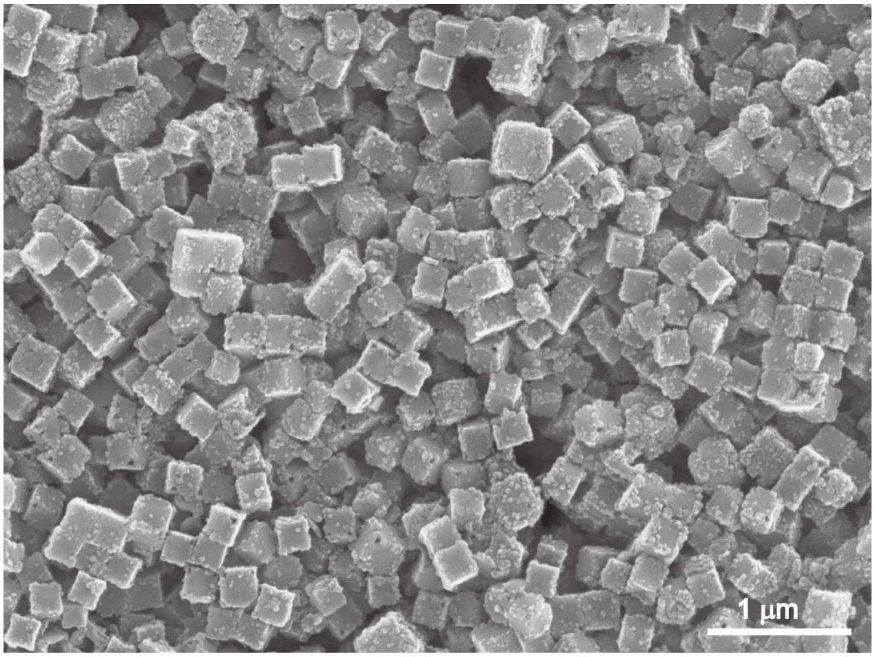

Figure S2. SEM image of CFHC.

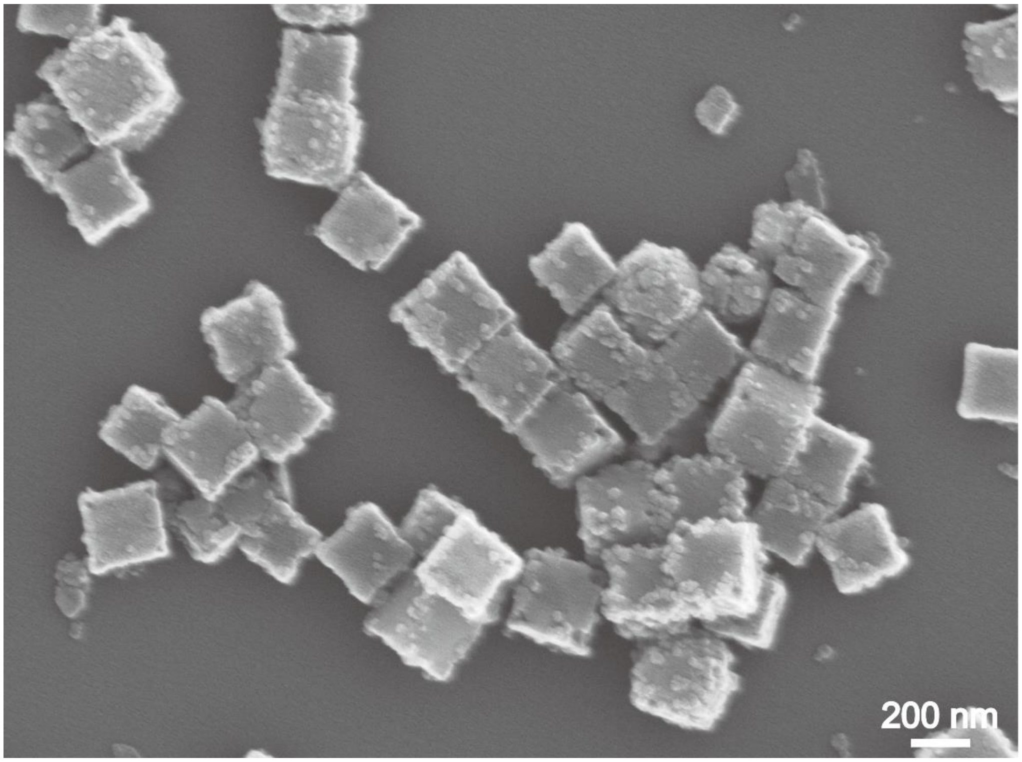

Figure S3. SEM image of CFHC.

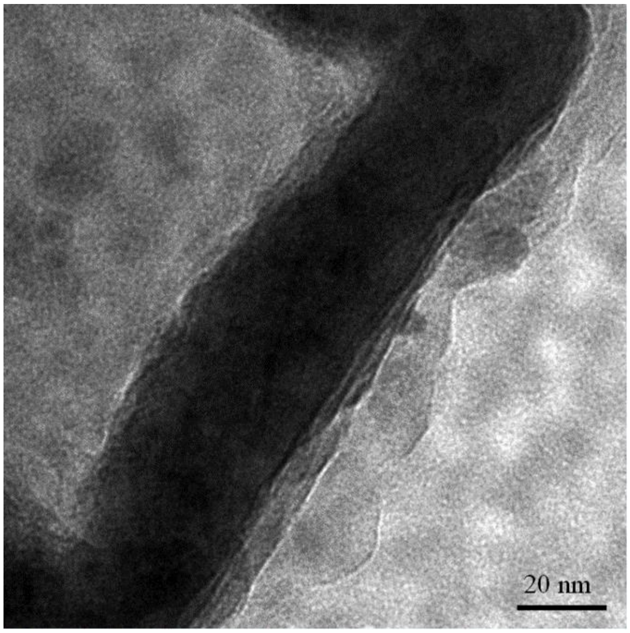

Figure S4.TEM image of CFHC.

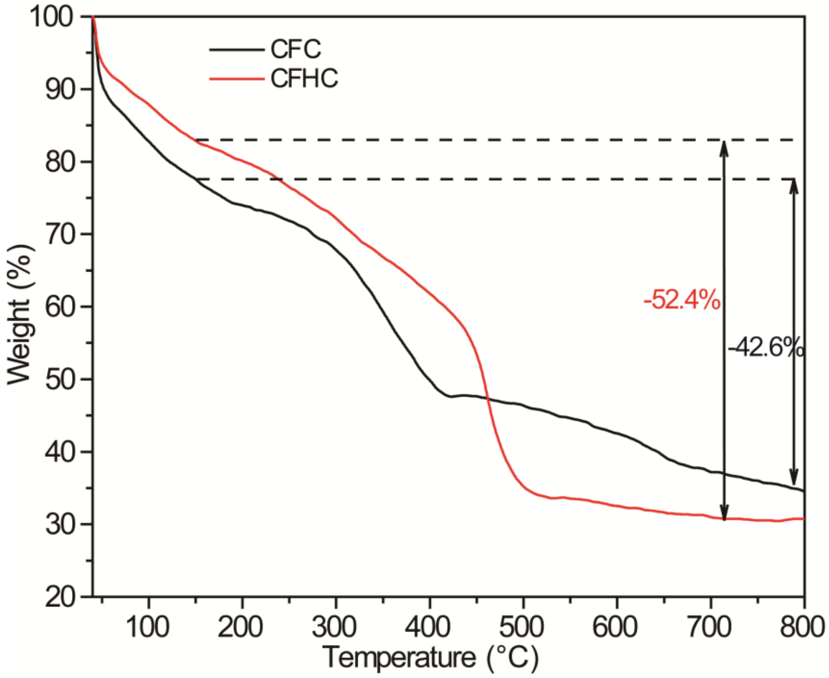

Figure S5. TGA pattern of CFHC (red) and CFC (black).

4. Synthesis and characterization of NFC and NFHC

NFC was synthesized using the similar method as CFC according to the literature.[1] In brief, $Ni(NO_3)_2{\cdot}6H_2O$ (0.6 mmol) and TCD (0.9 mmol) were dissolved in 20 mL of DI water to form solution A, and 0.4 mmol of $K_3[Fe(CN)_6]$ was added to another 20 mL of DI water to form solution B. Then solution A were poured into solution B under vigorous stirring. After several minutes, the mixtures were left to stand for 20 h without any disturbance. The thus-obtained CFC were collected by centrifugation at 8500 rpm for 15 min, followed by washing with water and EtOH three times, and then dried at 70 °C for 12 h. As can be seen from Fig. S6a,b, the as-synthesized NFC displayed a solid cubic morphology as its partner CFC with an average size of 200 nm.

The NFHC was synthesized through a solvothermal treatment. Experimentally, 20 mg of the as-synthesized NFC was dispersed in 20 mL of EtOH with the assistance of ultrasonication. This dispersion was then added into 20 mL of EtOH solution containing 100 mg of PVP under vigorously stirring. The obtained transparent solution was transferred to a Teflon-lined autoclave

capped with a stainless steel vessel. After heating at 180 ℃ for 24 h, the autoclave was cooled naturally to room temperature. The precipitates were collected by centrifuging at 8500 rpm for 15 min and washed with DI water and EtOH three times and dried at 70 ℃ for 12 h. As shown in Fig. S6c,d, the NFHC displayed a 3D hollow cubic cage structure. Different from CFHC, the interior of NFHC was not completely empty under the same solvothermal condition, indicating that NFHC underwent a slightly slower reaction kinetics. The HRTEM image shown in Fig. S6e illustrated a fringe crystal spacing of 0.518 nm, corresponding to the (200) plane of the typical PBA with a space group of $F\bar{4}3m$ (216).[2] SAED pattern in Fig. S6f clearly depicted the diffraction spots belong to (200), (400) and (422) planes.[3] Figure S6g showed the HAADF-TEM and the corresponding elemental mapping. It was noteworthy that the N and O distribution were a little larger than other elements, indicating that the PVP molecules were adsorbed on the surface of NFHC during the solvothermal reaction.

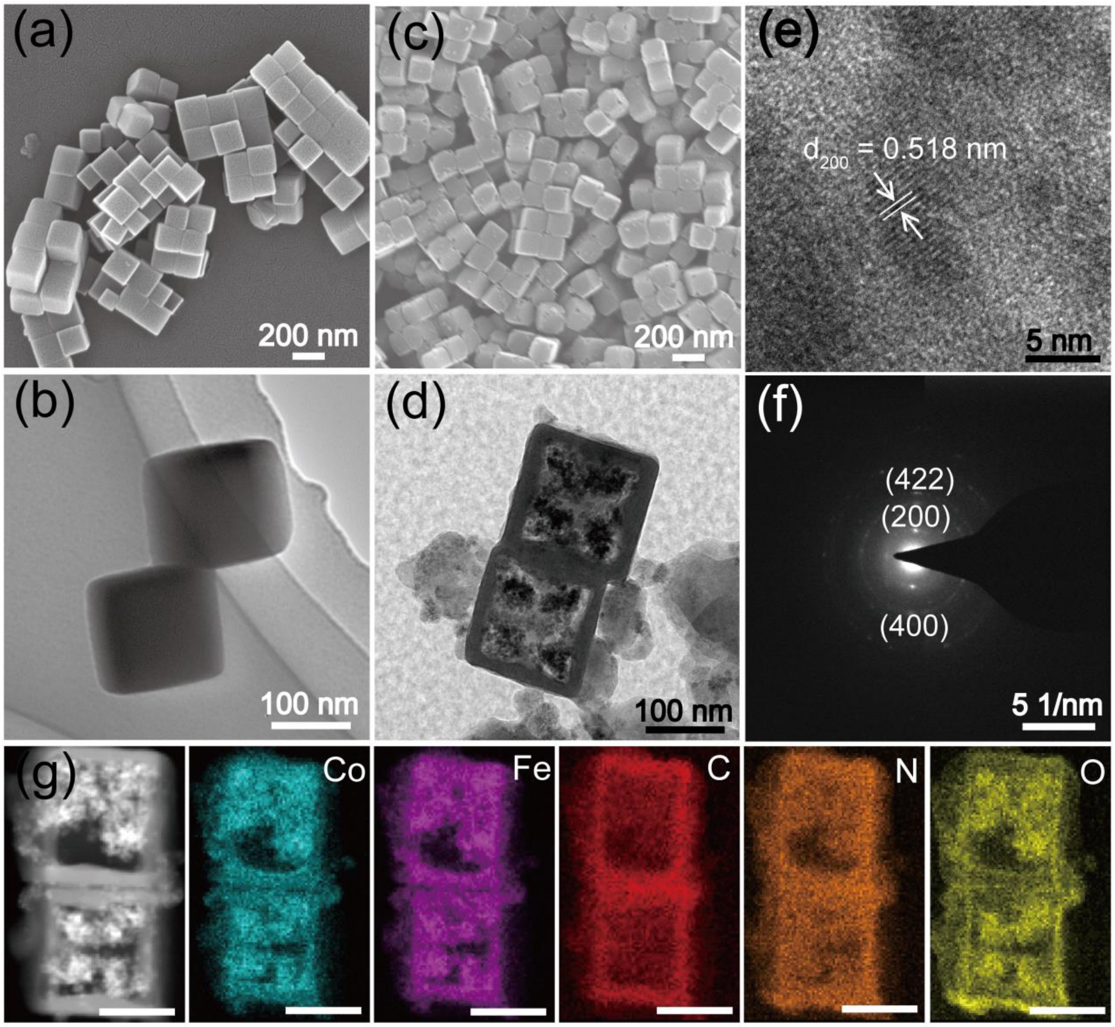

Figure S6. (a) SEM and (b) TEM images of NFC. (c) SEM and (d) TEM images of NFHC. (e) HRTEM and (f) SAED pattern of NFHC. (g) HAADF-TEM image of NFHC and the corresponding elemental mapping for Co (cyanide blue), Fe (purple), C (red), N (orange) and O (yellow), scale bar 100 nm.

5. Structure evolution of CFHC

Time-dependent SEM, TEM and XRD were performed to investigate the structure evolution of CFHC. As shown in Fig. S7, after solvothermal reaction for 1 h, the center part of the initial CFC started to dissolve and the cavity become larger along the body diagonal progressively. When the reaction time prolonged to 24 h, the interior become completely empty and the surface become

rough consisting of numerous Co-Fe PBA nanoparticles. Time-dependent XRD shown in Fig. S8, illustrated that with the reaction proceeding, the initial CFC underwent a structure contraction,[4-7] i.e., the cell parameter decreased from 10.295 Å in CFC to 10.269 Å in CFHC, which could be attributed to the bonding broken under the solvothermal reaction.

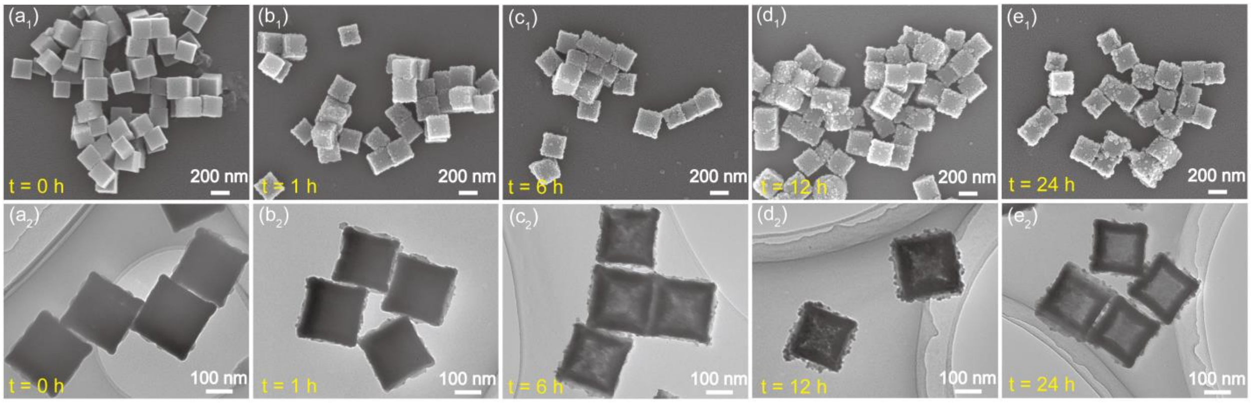

Figure S7. Time-dependent SEM (panel 1 of a-e) and TEM (panel 2 of a-e) images of CFHC at different reaction time, (a) 0 h, (b) 1 h, (c) 6 h and (d) 12 h, and (e) 24 h.

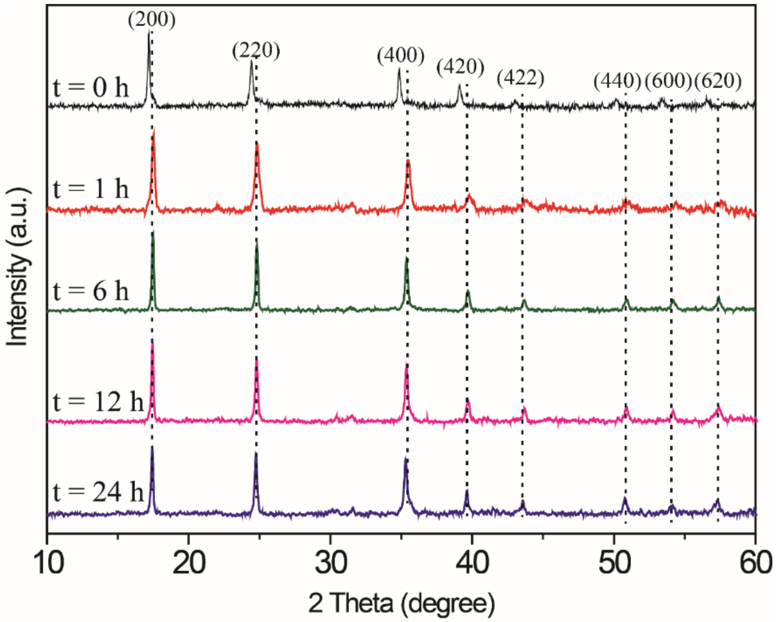

Figure S8. Time-dependent XRD patterns of CFHC at different reaction time. The dashed lines indicated the peak shift of CFHC relative to CFC.

The FTIR spectra of CFHC, NFHC along with CFC and NFC were shown in Fig. S9. The peak at 1607 and 1383 cm^{-1} in the FTIR spectrum of CFC shown in Fig. S9a were ascribed to vibration of –CN– in PBA frameworks, which were slightly upshifted after solvothermal treatment in CFHC. Notably, the peak located at 1293 cm^{-1} could be attributed to the –NC– in the amid bond of PVP, where the peak at 1412 cm^{-1} belonged to the –CH– in PVP, and peak at 1640 cm^{-1} was appointed to –CO– in PVP. These evidences demonstrated the fact the PVP moiety was chemically absorbed on the surface of CFHC during the solvothermal reaction. Similar result could be obtained in the FTIR spectra of NFHC as shown in Fig. S9b.

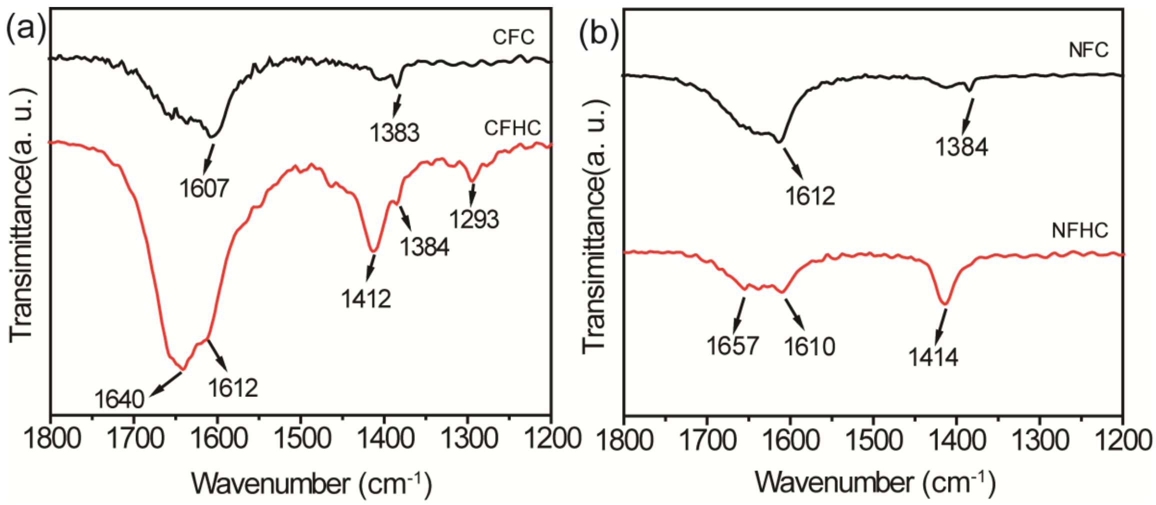

Figure S9. FTIR spectra of (a) CFC (black) and CFHC (red), and (b) NFC (black) and NFHC (red), respectively, in the range from 1200 to 1800 cm^{-1}.

6. Electronic Structure analysis of CFHC

XPS was conducted on CFHC and NFHC to unveil the electronic valence state after solvothermal treatment. Figure S10a,b illustrated the overlay spectra of CFHC and NFHC, respectively, confirming the elemental composition of the CFHC and NFHC. Similar to CFHC, in the high-resolution XPS profiles of Ni 2p, the main peaks located at 856.4 and 873.9 eV were ascribed to Ni^{3+} $2p_{3/2}$ and $2p_{1/2}$, respectively, and the binding energy at 857.9 and 875.4 eV were

assigned to Ni^{2+} $2p_{3/2}$.[1,8] Obviously, in NFHC the XPS peaks for Ni^{3+} were enhanced while the peak corresponding to Ni^{2+} was slightly decreased (Fig. S11a,c). Synchronously, the XPS peak for Fe^{3+} located at 708.2 eV was decreased and the peak for Fe^{2+} at 721.4 eV was increased (Fig. S11b,d).[1,9] Such valence change might be caused during the bonding broken while the solvothermal reaction. Notably, such charge transfer from Ni^{II}-CN-Fe^{III} → Ni^{III}-CN-Fe^{II} would facilitate the electrocatalytic process in the OER and UOR.

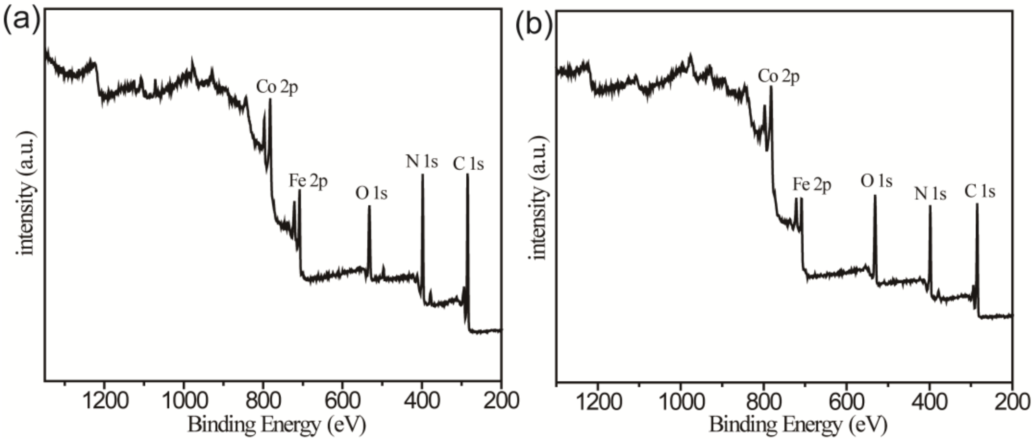

Figure S10. Overlay XPS spectra of CFHC (a) and NFHC (b).

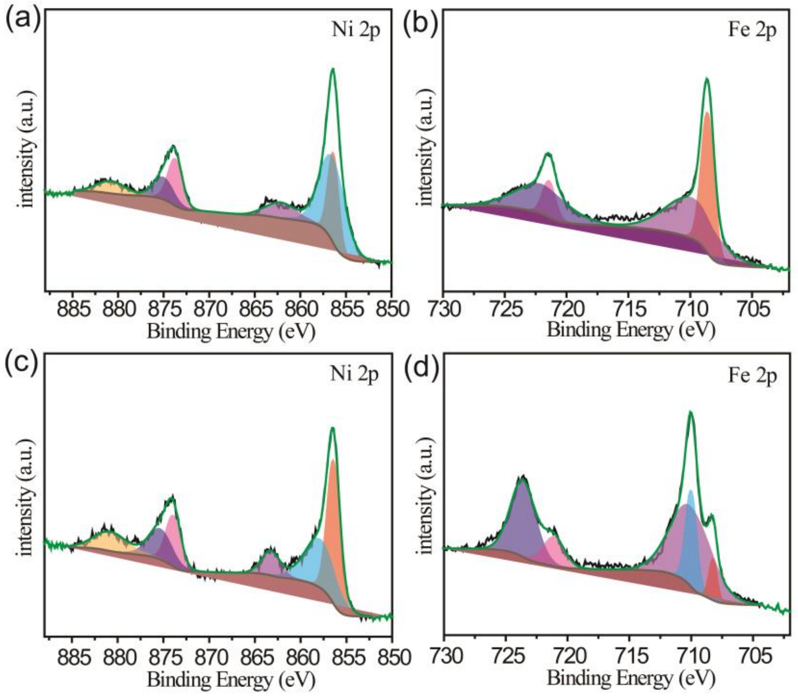

Figure S11. XPS spectra of Ni 2p (a ,c), Fe 2p (b, d) for NFC (a, b) and NFHC (c, d).

7. Possible mechanism for the formation of CFHC

To investigate the mechanism behind the solvothermal reaction for the formation of CFHC, a series of control experiments were carried out. Firstly, when the reaction temperature decreased to 160 °C, the cavity of the thus-obtained CFHC was not completely empty as shown in Fig. S12a,b, while the temperature increased to 200 °C, the so-obtained CFHC was almost collapsed (Fig. S12c,d), indicating that the reaction temperature was a vital factor to the formation of CFHC structure.

Another experiment demonstrated that PVP played an important role in the construction of hollow structure. As shown in Fig. S13, if no addition of PVP in the solvothermal reaction, the as-obtained CFHC become weak with a very thin cage shell, indicating that the surface adsorbed PVP molecules could protect the hollow structure and decrease the etching rate during the reaction.[10,11]

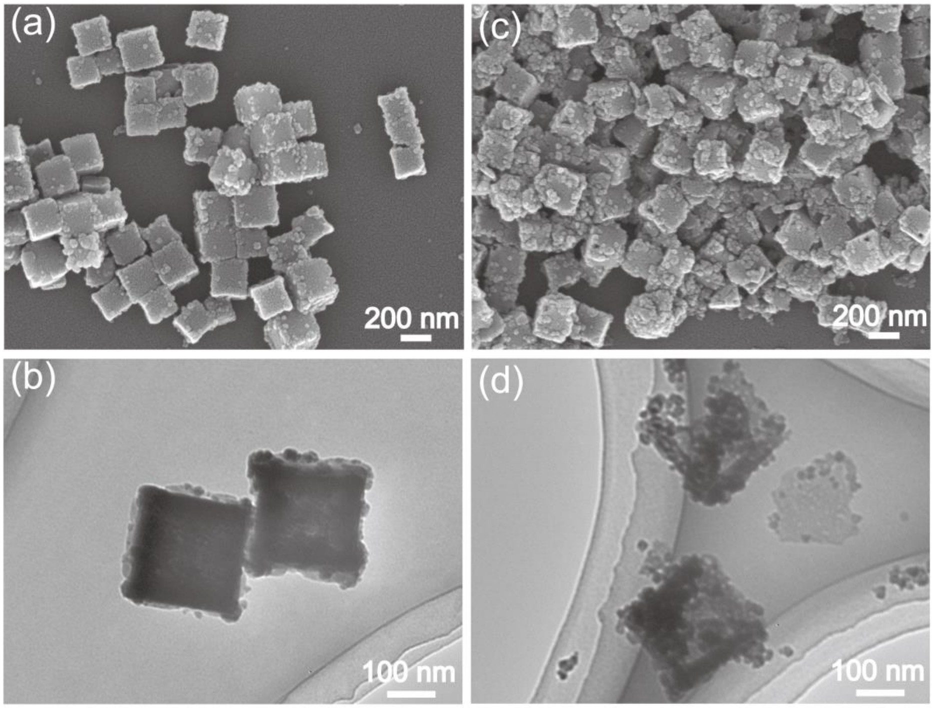

Figure S12. (a) SEM and (b) TEM images of CFHC, reaction condition: 100 mg PVP, 160 ℃, 24 h.(c) SEM and (d) TEM images of CFHC, reaction condition: 100 mg PVP, 200 ℃, 24 h.

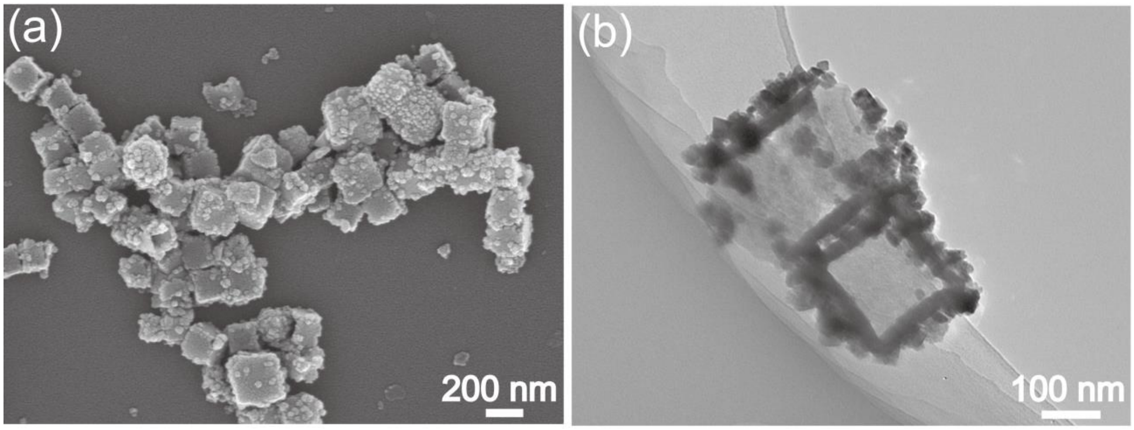

Figure S13. (a) SEM and (b) TEM images of CFHC, reaction condition: 0 mg PVP, 180 ℃, 24 h.

8. Electrocatalytic performance of CFHC and NFHC

The OER performance of NFHC were evaluated at the same condition as CFHC. As shown in Fig. S15a, the NFHC catalyst exhibited an overpotential of 313 mV to drive a current density of 20 mA cm^{-2}, lower than that of NFC (326 mV) and IrO_2 (388 mV). Even at higher current density, the NFHC still displayed lower overpotentials than its solid precursor NFC (Fig. S15b). The Tafel slope of NFHC, NFC and IrO_2 were 20, 92 and 78 mV dec^{-1}, respectively (Fig. S15c), indicating superior electrocatalytic kinetics of NFHC during OER process. Furthermore, the NFHC displayed smaller semi-circle in the EIS curve, which shown a charge transfer resistance of 14.36 Ω, much smaller than those of NFC (22.8Ω) and IrO_2 (54.2 Ω) (Fig. S15d). The double-layer capacitor (C_{dl}) of NFHC acquired from the CV scanning curves was 77.3 mF cm^{-2}, larger than that of NFC (18.2 mF cm^{-2}), indicative of larger electrochemical active surface area (ECSA) of NFHC (Fig. S16). Moreover, the CV test before and after 5000 continuousscanning without obvious change suggested the durability of the NFHC catalyst (Fig. S15e). Also, the long-term chronoamperometry test confirmed the stability of NFHC for at least 12 h with only slight decrease (Fig. S15f).

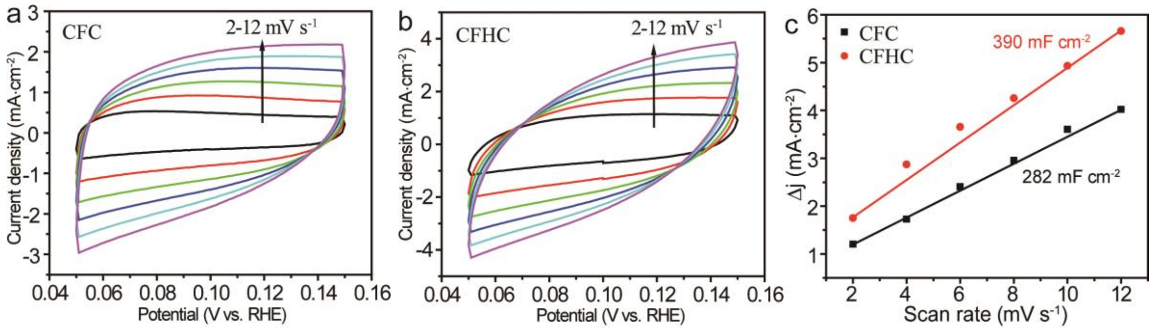

Figure S14. CV curves of (a) CFC and (b) CFHC with different scan rate from 2 to 12 mV in the range of 0.04-0.16 V in 1 M KOH, and (c) the corresponding current density difference at 0.10 V plot against the scan rate.

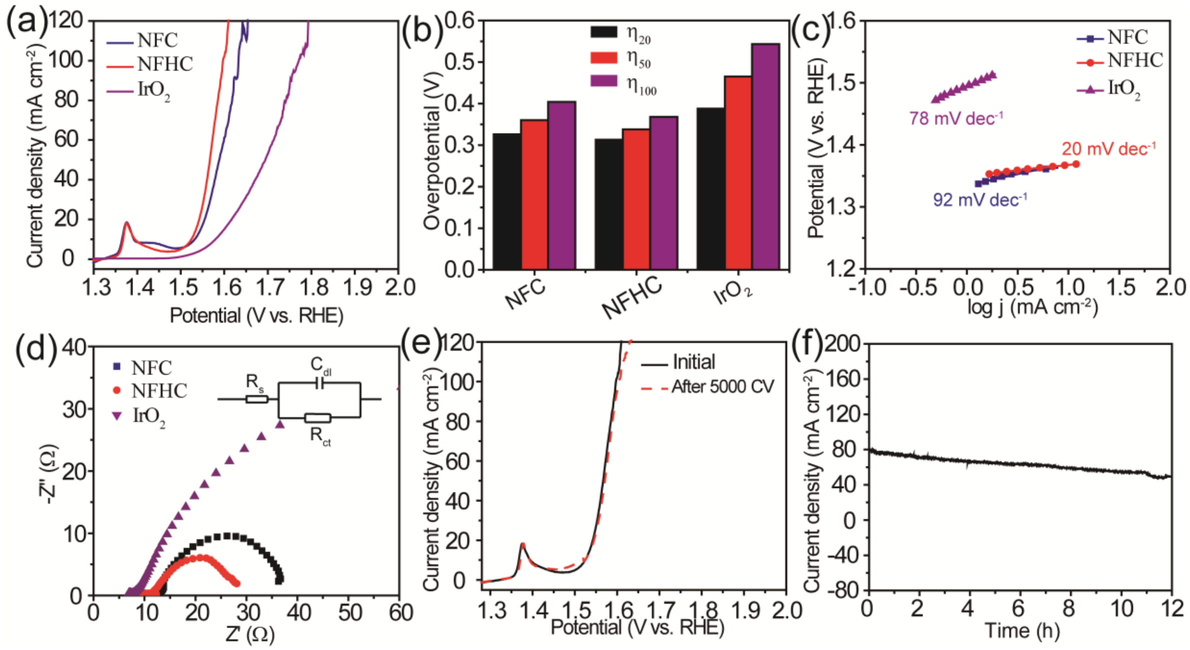

Figure S15. OER performance of the NFHC catalyst. (a) LSV curves of NFC, NFHC and IrO_2 and (b) their corresponding overpotentials showing η_{20}, η_{50}, η_{100} measured in 1 M KOH with a scan rate of 5 mV s^{-1}. (c) Tafel slopes and (d) EIS of NFC, NFHC and IrO_2, inset in d showing the equivalent circuit diagram. (e) LSV curves of NFHC before (solid black) and after (dashed red) 5000 CV cycles and (f) showing the i-t curve measured at 1.58 V.

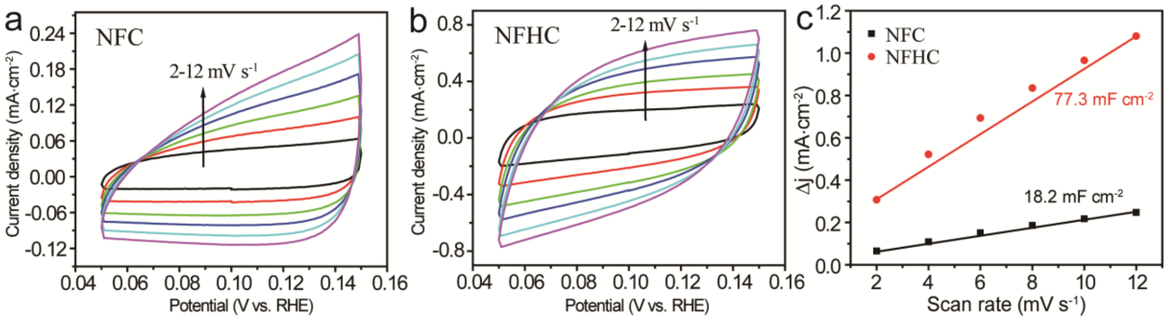

Figure S16. CV curves of (a) NFC and (b) NFHC with different scan rate from 2 to 12 mV in the range of 0.04-0.16 V in 1 M KOH, and (c) the corresponding current density difference at 0.10 V plot against the scan rate.

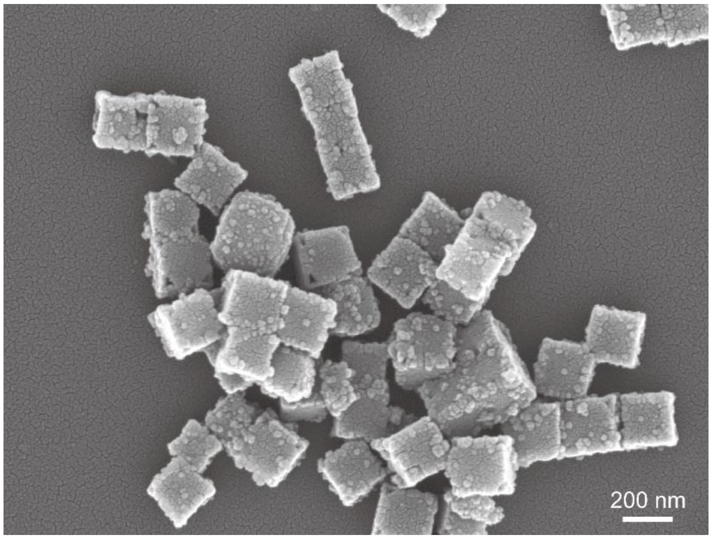

Figure S17. SEM image of CFHC after i-t measurement during the OER process.

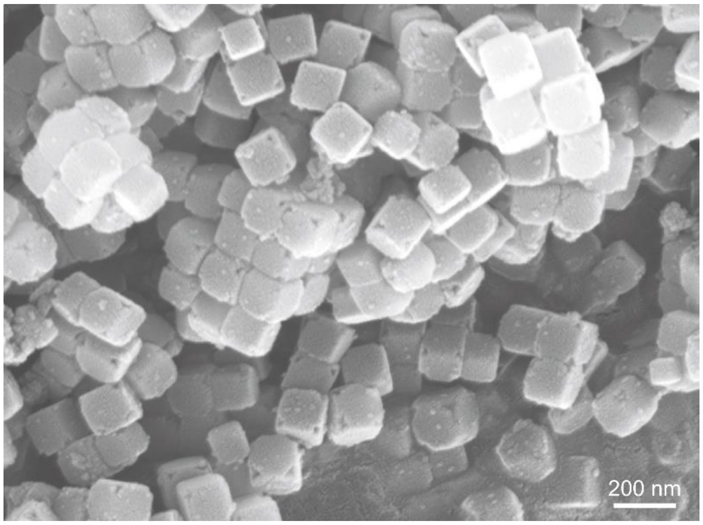

Figure S18. SEM image of NFHC after i-t measurement during the OER process.

The UOR performance of the CFHC catalyst was subsequently evaluated. As shown in Fig. S20a, the polarization curve of CFHC measured in 1 M KOH containing 0.5 M urea aqueous solution displayed an improved UOR performance compared with CFC and IrO_2. To deliver a current density of 10 mA cm^{-2}, the CFHC catalyst required a potential of 1.41 V, lower than those of CFC (1.45 V) and IrO_2 (1.49 V). The C_{dl} value of CFHC (507 mF cm^{-2}) was much larger than that of its counterpart CFC (228 mF cm^{-2}) (Fig. S21), demonstrating a favorable electrocatalytic activity of CFHC. The ECSA-normalized LSV curve shown in Fig. S22 confirmed the superior UOR behavior of CFHC. The Tafel slopes of CFHC, CFC and IrO_2 were 173, 217 and 420 mV dec^{-1} successively (Fig. S20b), indicating a faster catalytic kinetics of CFHC during the UOR process. Moreover, the R_{ct} value of CFHC was 23.7 Ω, much smaller than those of CFC (255.4 Ω) and IrO_2 (180.3 Ω) (Fig. S20c), implying that the CFHC catalyst underwent a favorable charge transfer process. Notably, the UOR performance of the CFHC catalyst was superior to the commercial IrO_2 and comparable to most previously reported non-precious metal-based catalysts (Table S2). In addition, the LSV curve after continuous CV scanning and long-term i-t test were

performed as shown in Fig. S20d. It was clear that after 5000 CV cycles, the LSV curve of the CFHC catalyst exhibited negligible change and the electrocatalytic activity can preserve at least for 12 h, indicative of a desirable catalytic stability.

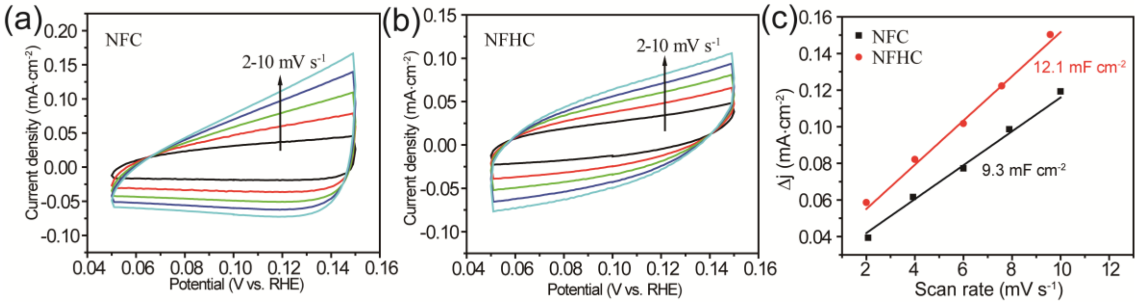

Figure S19. CV curves of (a) NFC and (b) NFHC with different scan rate from 2 to 10 mV in the range of 0.04-0.16 V in 1 M KOH containing 0.5 M urea, and (c) the corresponding current density difference at 0.10 V plot against the scan rate.

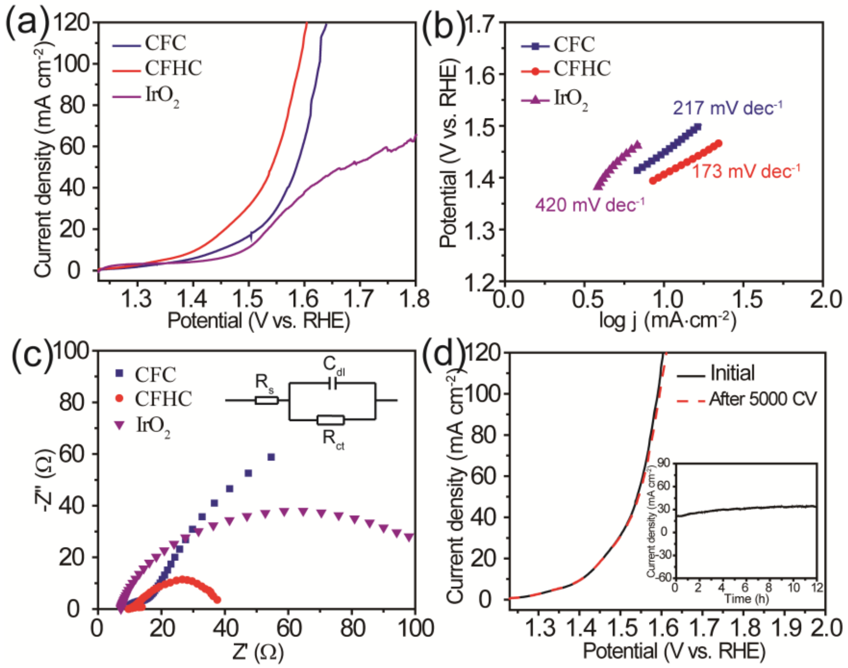

Figure S20. UOR performance of the CFHC catalyst. (a) LSV curves of CFC, CFHC and IrO_2 and (b) their corresponding Tafel slopesmeasured in 1 M KOH containing 0.5 M urea solution with a scan rate of 5 mV s^{-1}. (c) EIS of CFC, CFHC and IrO_2, inset in c showing the equivalent circuit

diagram. (d) LSV curves of CFHC before (solid black) and after (dashed red) 5000 CV cycles, inset showing the i-t curve measured at 1.46 V.

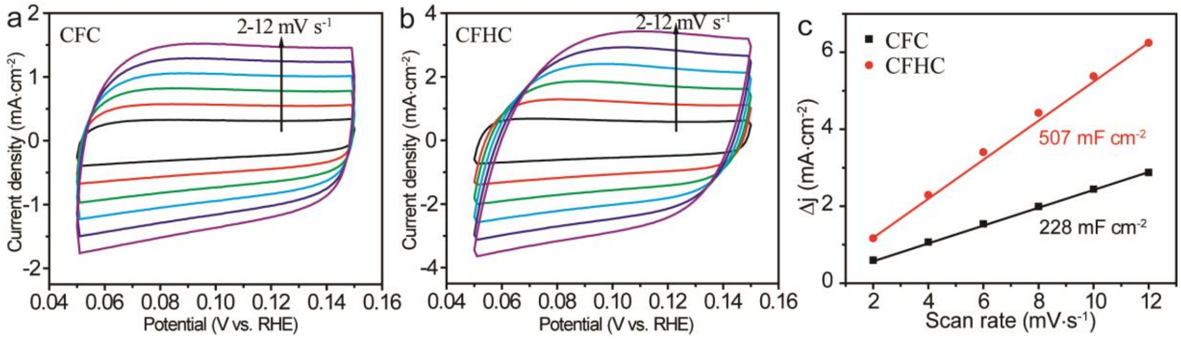

Figure S21. CV curves of (a) CFC and (b) CFHC with different scan rate from 2 to 12 mV in the range of 0.04-0.16 V in 1 M KOH containing 0.5 M urea, and (c) the corresponding current density difference at 0.10 V plot against the scan rate.

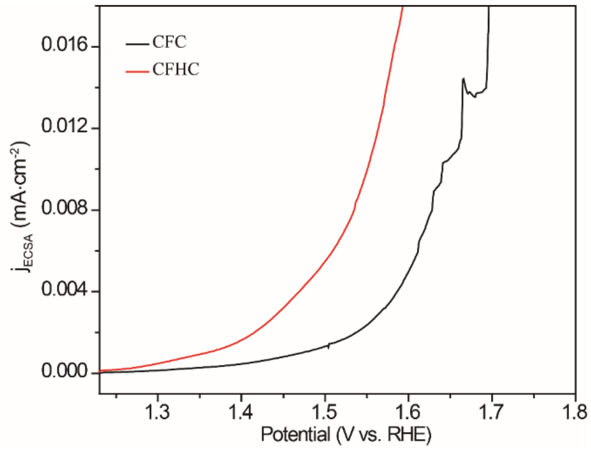

Figure S22. ECSA-normalized LSV curves of CFC and CFHC during UOR process.

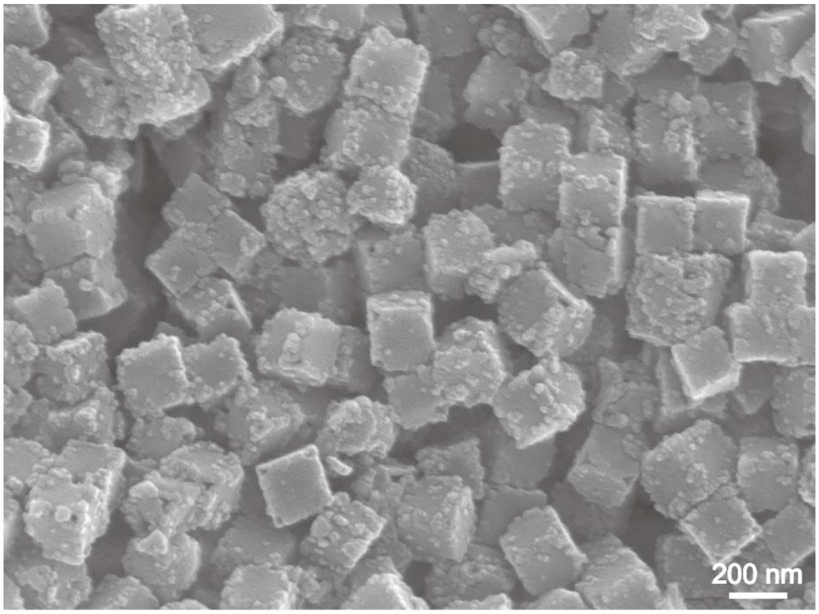

Figure S23. SEM image of CFHC after i-t measurement during the UOR process.

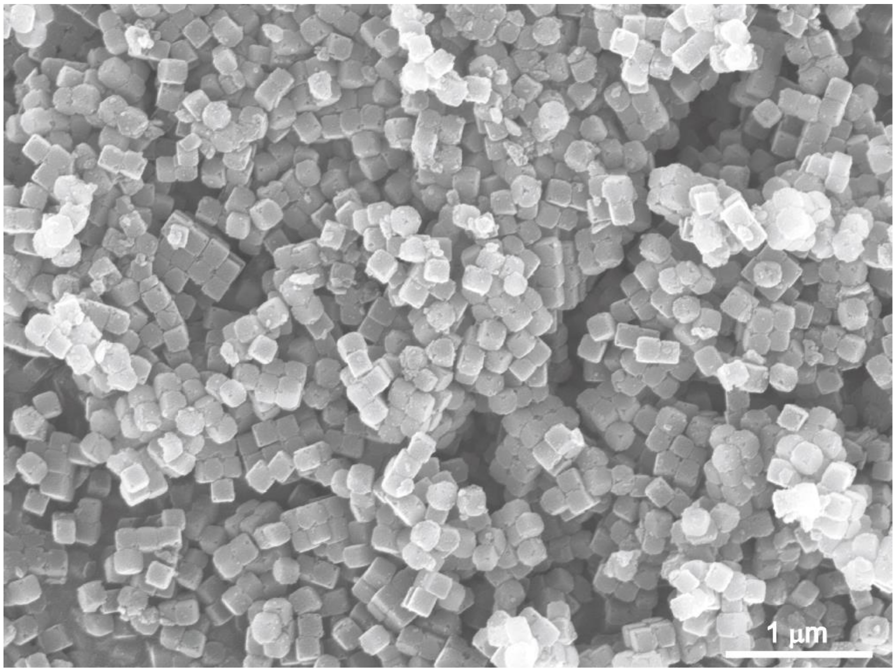

Figure S24. SEM image of NFHC after i-t measurement during the UOR process.

Table S1. The OER properties of CFHCand NFHC compared with other non-precious metal-based OER catalysts in 1 M KOH.

Catalysts	η_{10} (mV)	Tafel slope (mV dec^{-1})	Substrate	References
**CFHC**	330	57	Glassy carbon	This work
**NFHC**	313	20	Glassy carbon	This work
**CoMn-LDH**	324	43	Glassy carbon	J. Am. Chem. Soc. 2014, 136, 16481-16484
**(Ni,Co)Se_2-GA**	320	70	Nickel film	ACS Catal.2017, 79, 6394-6399
**Co-PBA-plasma**	274	53	Nickel film	Adv. Energy Mater. 2018, 8(18): 1800085
**NCF-MOF**	320[a]	49	Glassy carbon	Adv. Funct. Mater. 2018, 28, 1802129
**t-CoCo-PBA**	240	79	Carbon cloth	Angew. Chem. Int. Ed. 2018, 57, 1241 –1245
**Co-Fe-O frame**	290	62	Glassy carbon	Chem 2018, 4, 1-16
**Co_3O_4microframes**	370	53	Glassy carbon	Chem. Commun. 2016, 52, 6269-6272
**Ni-Co mixed oxide cages**	380	50	Glassy carbon	Adv. Mater. 2016, 28, 4601–4605
**$Fe_xCo_{1-x}OOH$**	266	30	Carbon fiber cloth	Angew. Chem. Int. Ed. 2018, 57, 2672-2676
**Co-Fe oxide NAFSs**	340	57	Glassy carbon	Sci. Adv. 2017, 3, e1700732
**Ni-Fe-P**	271	53	Glassy carbon	ACS Appl. Mater. Interfaces 2017, 9, 26134−26142
**NiCoP/C**	330	96	Glassy carbon	Angew. Chem. 2017, 129, 3955-3958
**Exfoliated NiCo LDH**	367	40	Glassy carbon	Nat. Commun. 2014, 5, 4477
**Co_3O_4-B nanosheets**	318	57.6	Glassy carbon	ACS Appl. Mater. Interfaces 2018, 10, 7079−7086
**Fe-NiCr_2O_4/NF**	228	57	Nickel film	Chem. Commun. 2018, 54, 5462-5465
**CoCN@PZS-450 ℃**	450	79	Glassy carbon	Sci China Mater 2018, 61, 686–696

[a] data were acquired in 0.1 M KOH.

Table S2. The UOR properties of CFHCand NFHC compared with other non-precious metal-based UOR catalysts in 1 M KOH with urea.

Catalysts	E_{10} (V)[a] @10 mA cm^{-2}	Substrate	References
**CFHC**	1.41<br>1.54 (E_{50})<br>1.59 (E_{100})	Glassy carbon	This work
**NFHC**	1.37<br>1.38 (E_{50})<br>1.40 (E_{100})	Glassy carbon	This work
**NF-G-Mn**	1.33	Nickel foam	Angew. Chem. Int. Ed. 2016, 55, 3804-3808
**$NiCo_2O_4$**	1.77(E_{136})	stainless steel mesh	Nanoscale 2014, 6, 1369-1376
**Ni-MOF**	1.37	Glassy carbon	Chem. Commun. 2017, 53, 10906-10909
**Metallic $Ni(OH)_2$**	1.39*	Glassy carbon	Angew. Chem. Int. Ed. 2016, 55, 12465-12469
**NF/NiMoO-Ar**	1.37	Nickel foam	Energy Environ. Sci. 2018, 11, 1890-1897
**$Ni_{0.67}Co_{0.33}(OH)_2$/CC**	1.23	Carbon cloth	Nanoscale 2018, 10, 21087-21095
**$Ni(OH)_2$ NS@NW/NF**	1.41	Nickel foam	ElectrochimicaActa 2018, 268, 211-217
**$Fe_{11.1\%}$-Ni_3S_2/Ni foam**	1.35	Nickel foam	J. Mater. Chem. A 2018, 6, 4346-4353
**Ni_3NNA/CC**	1.35	Carbon cloth	Inorg. Chem. Front. 2017, 4, 1120-1124
**Ni_2P NF/CC**	1.46 (E_{60})	Carbon cloth	J. Mater. Chem. A 2017, 5, 3208-3213
**MnO_2/$MnCo_2O_4$/Ni**	1.7 (E_{386})	Nickel foam	J. Mater. Chem. A, 2017, 5, 7825-7832
**$NiCo_2S_4$ NS/CC**	1.32	Carbon cloth	ACS Sustainable Chem. Eng. 2018, 6, 5011-5020

[a]E_j refers to the potential required at current density j (mA cm^{-2}). * value calculated from the data in the literature.

9. Reference

1 Feng, Y., Sakaki, M., Kim, J.-h., Huang, J. & Kajiyoshi, K. Novel Prussian-blue-analogue microcuboid assemblies and their derived catalytic performance for effective reduction of 4-nitrophenol. *New Journal of Chemistry* **42**, 20212-20218 (2018).

2 Nai, J., Zhang, J. & Lou, X. W. D. Construction of single-crystalline Prussian blue analog hollow nanostructures with tailorable topologies. *Chem* **4**, 1967-1982 (2018).

3 Nai, J., Guan, B. Y., Yu, L. & Lou, X. W. D. Oriented assembly of anisotropic nanoparticles into frame-like superstructures. *Science advances* **3**, e1700732 (2017).

4 Higel, P., Villain, F. o., Verdaguer, M., Rivière, E. & Bleuzen, A. Solid-state magnetic switching triggered by proton-coupled electron-transfer assisted by long-distance proton-alkali cation transport. *Journal of the American Chemical Society* **136**, 6231-6234 (2014).

5 Bleuzen, A. *et al.* Thermally Induced Electron Transfer in a CsCoFe Prussian Blue Derivative: The Specific Role of the Alkali‐Metal Ion. *Angewandte Chemie International Edition* **43**, 3728-3731 (2004).

6 Goberna-Ferrón, S., Hernández, W. Y., Rodríguez-García, B. & Galán-Mascarós, J. R. n. Light-driven water oxidation with metal hexacyanometallate heterogeneous catalysts. *ACS catalysis* **4**, 1637-1641 (2014).

7 Felts, A. C. *et al.* Evidence for Interface-Induced Strain and Its Influence on Photomagnetism in Prussian Blue Analogue Core–Shell Heterostructures, $Rb_aCo_b[Fe(CN)_6]_c \cdot mH_2O@K_jNi_k[Cr(CN)_6]_l \cdot nH_2O$. *The Journal of Physical Chemistry C* **120**, 5420-5429 (2016).

8 Yu, X. Y. *et al.* Formation of Ni–Co–MoS_2 nanoboxes with enhanced electrocatalytic activity for hydrogen evolution. *Advanced Materials* **28**, 9006-9011 (2016).

9 Guo, Y. *et al.* Air Plasma Activation of Catalytic Sites in a Metal‐Cyanide Framework for Efficient

Oxygen Evolution Reaction. *Advanced Energy Materials* **8**, 1800085 (2018).

10 Hu, M., Belik, A. A., Imura, M. & Yamauchi, Y. Tailored design of multiple nanoarchitectures in metal-cyanide hybrid coordination polymers. *Journal of the American Chemical Society* **135**, 384-391 (2012).

11 Hu, M. *et al*. Synthesis of Prussian blue nanoparticles with a hollow interior by controlled chemical etching. *Angewandte Chemie International Edition* **51**, 984-988 (2012).

Address correspondence to Yongqiang Feng, fengyq@sust.edu.cn; Jianfeng Huang, huangjf@sust.edu.cn. Chunru Wang, crwang@iccas.ac.cn
